# From Polyphenols to Prodrugs: Bridging the Blood–Brain Barrier with Nanomedicine and Neurotherapeutics

**DOI:** 10.3390/ijms27052370

**Published:** 2026-03-03

**Authors:** Masaru Tanaka, Adriano Cressoni Araujo, Vítor Engrácia Valenti, Elen Landgraf Guiguer, Vitor Cavallari Strozze Catharin, Cristiano Machado Gualhardi, Eliana de Souza Bastos Mazuqueli Pereira, Ricardo de Alvares Goulart, Rafael Santos de Argolo Haber, Atonelly Cassio Alves de Carvalho, Sandra Maria Barbalho

**Affiliations:** 1 Danube Neuroscience Research Laboratory, HUN-REN-SZTE Neuroscience Research Group, Hungarian Research Network, University of Szeged (HUN-REN-SZTE), H-6725 Szeged, Hungary; 2 Department of Biochemistry and Pharmacology, School of Medicine, Faculdade de Medicina de Marília, Universidade de Marília (UNIMAR), Marilia 17525-902, SP, Brazilvitorcavallaristrozzecatharin@gmail.com (V.C.S.C.);; 3 Graduate Program in Structural and Functional Interactions in Rehabilitation, School of Medicine, Universidade de Marília (UNIMAR), Marilia 17525-902, SP, Brazil; 4 Systematic Reviews and Meta-Analyses Center, School of Philosophy and Sciences, São Paulo State University, Marilia 17525-900, SP, Brazil; 5 Research Coordination, UNIMAR Charity Hospital, Faculdade de Medicina de Marília, Universidade de Marília (UNIMAR), Marilia 17525-902, SP, Brazil

**Keywords:** central nervous system diseases (CNS), blood–brain barrier (BBB), drug delivery systems, nanomedicine, phytochemicals, nanoparticles, prodrugs, kynurenines, intranasal, ultrasonic therapy, transferrin receptor

## Abstract

Central nervous system disorders drive disability, yet many neuroactive candidates fail because the brain is a hard compartment to dose. Plant-derived molecules spanning polyphenols, alkaloids, terpenoids, and cannabinoids are attractive because their pleiotropic actions can engage oxidative stress, neuroinflammation, and circuit dysfunction. In practice, the blood–brain barrier (BBB) restricts most native phytochemicals through tight-junction selectivity, rapid metabolism, low solubility, and transporter-mediated efflux. Key gaps include poor standardization of exposure metrics, limited human-relevant BBB models, and few head-to-head studies that compare delivery platforms on the same payload and outcome. This review tackles the mismatch between mechanistic promise and reliable brain exposure that stalls translation. The objectives are to link phytochemical liabilities to enabling strategies in nanomedicine, alternative routes, and transporter-targeted prodrugs, and to propose decision-grade endpoints for translation. We synthesize evidence on BBB transport logic, nanocarrier families, targeting ligands, intranasal delivery, focused ultrasound-mediated opening, and prodrug approaches that hijack influx transporters, while foregrounding safety and chemistry, manufacturing, and controls (CMC) constraints. Here we highlight that effective neurotherapeutics emerge when chemistry, carrier, route, and measurement are co-designed rather than optimized in isolation. This framework can guide platform selection, de-risk first in-human studies, and sharpen trial endpoints. More broadly, it offers a transferable playbook for barrier-limited drug development across neurology, psychiatry, and oncology.

## 1. Introduction

### 1.1. Clinical Burden and Therapeutic Gap

Central nervous system (CNS) disorders such as depression, dementia, and chronic pain remain among the leading causes of global morbidity, disability, and economic burden [1,2]. Despite decades of intensive research, therapeutic outcomes remain unsatisfactory, with high relapse rates in major depressive disorder, limited disease-modifying options for dementia, and inadequate pain control across populations [2,3]. These shortcomings are amplified by the fact that most CNS-active drugs show poor penetration across the blood–brain barrier (BBB), resulting in suboptimal central exposure and attenuated efficacy [1,4]. Even when new compounds demonstrate preclinical promise, attrition rates during clinical translation remain staggering, with failure rates in neuropsychiatric drug development exceeding those in nearly all other therapeutic domains [5,6]. The net result is a widening therapeutic gap that leaves millions of patients reliant on outdated, partially effective, or poorly tolerated interventions [2,5,6,7].

This persistent impasse has renewed attention toward alternative sources of therapeutic innovation. Plant-derived molecules, particularly those rooted in neuroactive amino acid metabolism such as tryptophan, offer a compelling avenue [8,9,10,11,12]. These compounds are celebrated for their structural diversity, multitarget activity, and evolutionary compatibility with human physiology, making them attractive candidates for modulating complex CNS pathologies [8,10,13]. Yet, enthusiasm is tempered by major barriers. Many phytochemicals exhibit low bioavailability, poor stability, and unpredictable BBB permeability, which compromise their therapeutic impact [8,13,14,15]. Recent advances in nanotechnology, ranging from functionalized nanoparticles to receptor-assisted carriers, seek to overcome these pharmacokinetic and delivery hurdles, but their clinical translation is still in its infancy [8,13,14,15]. Against this backdrop, revisiting plant-derived tryptophan and its metabolic derivatives provides a unique opportunity to bridge neurobiology and psychiatry, while also testing the integration of phytochemistry with advanced delivery platforms to transform depression management [8,9,10,14,16].

### 1.2. Blood–Brain Barrier (BBB) as a Bottleneck for Phytochemicals

The BBB stands as the central checkpoint governing molecular access to the brain, designed to maintain homeostasis while excluding xenobiotics and potential toxins [13,17]. Its architecture is highly specialized: endothelial cells form continuous tight junctions that restrict paracellular flux, while efflux transporters such as P-glycoprotein (P-gp), breast cancer resistance protein, and multidrug resistance-associated proteins actively pump out diverse substrates [17]. This dual protection ensures neural integrity but also severely limits drug delivery [18]. For therapeutic compounds to penetrate effectively, they must navigate a gauntlet of physicochemical constraints. Molecules with poor aqueous solubility, inappropriate lipophilicity, or rapid metabolic breakdown are particularly disadvantaged, leading to negligible CNS exposure despite robust systemic availability [18,19]. Among phytochemicals, polyphenols have drawn particular attention due to their broad bioactivity and consistent links to neuroprotective and anti-inflammatory pathways [20,21]. Accordingly, this review is organized around three phytochemical classes relevant to brain delivery, polyphenols, alkaloids, and terpenoids plus cannabinoids. We discuss each group through the lens of BBB exposure constraints and the downstream neuroprotective and anti-inflammatory mechanisms they may engage.

Phytochemicals exemplify this paradox. Polyphenols such as resveratrol and curcumin exhibit potent antioxidant and neuroprotective activities in vitro, yet their hydrophilicity and metabolic instability limit their brain levels to trace amounts [13,15,22,23,24,25,26,27]. Alkaloids are introduced as a contrast class because they often appear more permeable based on lipophilicity, yet transporter recognition and efflux can still keep CNS exposure low [28]. Alkaloids, though often more lipophilic, encounter substantial efflux clearance, which nullifies their apparent permeability advantage [13]. Terpenoids and cannabinoids are not polyphenols. We include them here to show that high lipophilicity can still fail to translate into reliable brain exposure because metabolism and efflux remain dominant constraints [28,29]. Terpenoids and cannabinoids, despite their lipophilic structures, which favor passive diffusion, are hindered by rapid first-pass metabolism and limited bioavailability, yielding inconsistent central effects [15,22,30]. Studies consistently report that only a minority of native phytochemicals achieve detectable brain penetration, and even fewer reach concentrations required for therapeutic modulation of neurotransmission or neuroinflammation [13,31,32]. The recurring outcome is a stark disconnect between preclinical promise and clinical translation [22,33,34]. These limitations underscore the need for innovative delivery strategies that can re-engineer phytochemicals to evade efflux, improve stability, and optimize solubility [13,15,30,33]. Without such advances, the native molecular forms of these plant-derived agents remain ill-suited for reliable CNS targeting and fall short of their therapeutic potential in depression and related disorders [35,36].

### 1.3. Scope and Organizing Framework

This review is not a catalog of every BBB nanotechnology reported to date. Instead, we use an organizing framework that starts with payload liabilities, such as poor stability, rapid clearance, limited permeability, or off-target exposure, and then maps these constraints onto enabling strategies, from ligand-targeted carriers and intranasal systems to transporter-leveraging prodrugs and selected physical modulation [37,38,39]. We then judge platforms by pharmacological endpoints that matter for CNS translation: quantifiable brain exposure, target engagement, and a safety margin compatible with real world dosing [37,40,41,42].

Accordingly, we largely exclude systemic nanomedicine programs without explicit CNS intent, purely diagnostic nanomaterials, and highly speculative constructs lacking a plausible CMC and regulatory path. With that scope set, the next step is to ground these choices in the biological rules of the barrier itself, because delivery design only works when it respects architecture, transport routes, and disease-driven heterogeneity. To keep the narrative cohesive, we proceed from barrier biology to actionable delivery decisions. Section 2 summarizes BBB architecture, transport routes, and disease-driven heterogeneity that shape access to the brain. Section 3, Section 4, Section 5 and Section 6 then profile phytochemical classes, their key liabilities, and the enabling toolbox spanning nanocarriers, targeting ligands, and responsive or route-based strategies. Section 7, Section 8, Section 9, Section 10, Section 11 and Section 12 focus on measurement and translation, including exposure metrics, model selection, clinical signals, and decision grade endpoints. Finally, Section 13, Section 14 and Section 15 synthesize short-term development priorities, CMC constraints, and a pragmatic roadmap for moving phytochemicals toward testable neurotherapeutics.

## 2. The Blood–Brain Barrier (BBB): Architecture, Transport, Heterogeneity

This section lays out the BBB features that matter most when you are trying to move neuroactive compounds from blood to brain. We first describe the neurovascular unit as the physical and regulatory scaffold that sets baseline permeability. Next, we map the main transport routes, from carrier and receptor-mediated entry to the efflux pumps that push xenobiotics back out. Finally, we highlight BBB heterogeneity across regions, aging, and disease, because delivery rules change when the barrier itself changes.

### 2.1. Neurovascular Unit Architecture

The neurovascular unit (NVU) forms the structural and functional foundation of the BBB, integrating multiple cellular and extracellular components into a finely tuned system that maintains CNS homeostasis [43,44,45]. Far from being a passive wall, the NVU is a dynamic interface whose architecture underpins both the protective selectivity of the barrier and its vulnerability in disease states [44,46,47].

At the core of this architecture are brain microvascular endothelial cells, which adopt a highly specialized phenotype distinct from systemic endothelia [44,48]. They exhibit extremely low rates of transcytosis and lack fenestrations, thereby minimizing nonspecific permeability [48]. Their intercellular contacts are enriched with tight junction proteins such as claudins, occludin, and ZO-1, creating an electrically resistant barrier that restricts paracellular diffusion while still permitting finely regulated transport of essential metabolites [49,50,51]. Adherens and gap junctions provide additional stability and communication, allowing endothelial cells to operate as a synchronized sheet rather than as isolated units [50,52].

Closely apposed to the endothelial layer, pericytes are embedded within the basement membrane and act as guardians of barrier integrity [47,53]. They regulate angiogenesis, modulate permeability, and secrete trophic factors such as angiopoietin I and vitronectin, which sustain endothelial survival and limit inflammatory activation [54]. Astrocytic endfeet ensheath nearly the entire capillary surface, releasing mediators, including vascular endothelial growth factor (VEGF) and glial-derived neurotrophic factor (GDNF), while their aquaporin-4 channels orchestrate water and ion balance, which are crucial for neuronal signaling [43,48,55,56].

The basement membrane itself, composed of extracellular matrix proteins secreted by both endothelial cells and astrocytes, provides not only structural stability but also biochemical cues that regulate cellular behavior and crosstalk within the NVU [44,57]. Central to barrier impermeability are tight and adherens junctions, which act as molecular rivets sealing adjacent endothelial cells [49,50]. These complexes are remarkably plastic, responding to oxidative stress, inflammation, and neurodegenerative insults by loosening or disassembling, thereby amplifying barrier leakiness [49,51,53]. Altogether, the NVU’s intricate cellular and extracellular architecture forms the scaffold upon which selective transport processes are built, ensuring both the protection and the metabolic supply of the CNS [43,44,45].

### 2.2. Transport Pathways and Efflux

The BBB operates under the constant tension of permitting the entry of essential nutrients while simultaneously excluding xenobiotics and potentially harmful agents. This balancing act defines its role as both protector and barrier, a duality that complicates CNS drug development [4,58,59]. Only a limited fraction of compounds traverses the BBB by passive diffusion, largely restricted to small, lipophilic molecules with low molecular weight [4,58,60]. Even for lipophilic drugs, passage is often curtailed by additional regulatory mechanisms that actively limit nonspecific entry [58,61].

**Figure 1:**
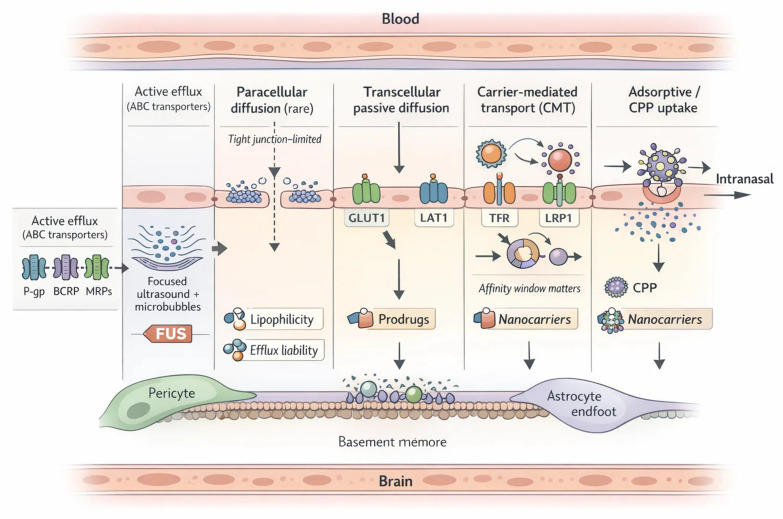
Blood–brain barrier (BBB) transport routes and where delivery platforms intervene: passive limits, engineered influx, and efflux ceilings. The schematic summarizes dominant BBB transport routes relevant to phytochemicals and highlights where delivery platforms intervene. At an intact BBB, paracellular diffusion across tight junctions is negligible and disease-dependent; controlled opening is best treated as a localized modulation strategy (e.g., focused ultrasound (FUS) with microbubbles) rather than a baseline assumption. Transcellular passive diffusion depends on size, polarity, hydrogen bonding, and lipophilicity, but apparent permeability is often capped by metabolism and active efflux. ABC transporters—P-gp, BCRP, MRPs—frequently set the ceiling for unbound brain exposure, motivating efflux-evading prodrugs, stealth/corona control for nanocarriers, and early efflux-liability screening. For polar phytochemicals, carrier-mediated transport (CMT) via GLUT1, LAT1, and MCT1 enables engineered influx using nutrient-mimetic promoieties, requiring K_m_/V_MAX_-aware design and brain-selective cleavage. For larger cargos, receptor-mediated transcytosis (RMT) via TfR and LRP1 supports ligand-decorated nanocarriers, but demands an affinity “sweet spot” to avoid endothelial sequestration and lysosomal routing. Adsorptive/CPP uptake can boost entry yet trades specificity for off-target risk; charge-switchable or stimulus-unmasked coatings can mitigate this. Intranasal delivery provides a complementary bypass for suitable payloads. ABC, ATP-binding cassette; BBB, blood–brain barrier; BCRP, breast cancer resistance protein; CMT, carrier-mediated transport; CPP, cell-penetrating peptide; FUS, focused ultrasound; GLUT1, glucose transporter 1; K_m_, Michaelis constant; LAT1, L-type amino acid transporter 1; LRP1, low-density lipoprotein receptor-related protein 1; MCT1, monocarboxylate transporter 1; MRPs, multidrug resistance-associated proteins; P-gp, P-glycoprotein; RMT, receptor-mediated transcytosis; TfR, transferrin receptor; V_MAX_, maximum transport rate.

To sustain brain metabolism, the BBB relies heavily on carrier-mediated transport [4,60]. Prominent examples include GLUT1 for glucose, LAT1 for large neutral amino acids, and monocarboxylate transporters (MCTs) for lactate and other energy substrates [61,62]. These carriers not only ensure nutrient delivery but also provide entry routes for select phytochemicals, though their activity is sensitive to pathological states and drug interactions [61,62]. For larger molecules, receptor-mediated transcytosis represents a critical pathway, with transferrin and insulin receptors serving as canonical examples [63,64]. Advances in nanomedicine are increasingly focused on exploiting these receptors to deliver therapeutic payloads across the barrier in a controlled manner [58,59,65]. Opposing these influx mechanisms are efflux pumps, the most formidable being P-gp, breast cancer resistance protein (BCRP), and multidrug resistance-associated proteins (MRPs) [58,61]. These ATP-binding cassette (ABC) transporters expel a vast array of xenobiotics and pharmacological compounds, shaping drug distribution within the brain [58,61]. Their expression is dynamic, influenced by neuronal activity, circadian rhythms, aging, and disease states, while interactions with polyphenols and phytochemicals may either inhibit or stimulate efflux, thereby altering drug bioavailability [4]. Ultimately, BBB transport functions as both a sentinel and a bottleneck, safeguarding the CNS but at the same time restricting the therapeutic reach of many promising neuroactive agents [58,59,66] (Table 1, Figure 1).

### 2.3. Disease- and Age-Driven Heterogeneity

The BBB is not a uniform structure but displays striking regional heterogeneity that shapes vulnerability and therapeutic access [78,79,80]. The hippocampus, for example, exhibits earlier and more pronounced permeability changes compared to the cortex, while the choroid plexus contains fenestrated vasculature that facilitates selective exchange with cerebrospinal fluid [78,80,81,82,83]. Such regional differences are further accentuated by pathological states: aging, neurodegeneration, and systemic inflammation remodel barrier integrity, producing spatially distinct patterns of leakage and dysfunction [80,84,85,86,87]. These dynamic alterations contribute to selective regional susceptibility in disorders such as Alzheimer’s and Parkinson’s disease [80,84,86,88]. Recognizing and integrating BBB heterogeneity is therefore critical for the rational design of nanomedicine and targeted neurotherapeutics [4,58,89,90,91].

Ultimately, BBB transport functions as both a sentinel and a bottleneck, safeguarding the CNS but at the same time restricting the therapeutic reach of many promising neuroactive agents [4,58,89,90,91]. Building on this transport landscape, the subsequent section turns to phytochemicals, profiling their distinct liabilities and highlighting how these molecular features dictate both formulation choices and prodrug design in the pursuit of effective neurotherapeutics [14,15,92,93]. Section 3 is organized by phytochemical class, since each class tends to hit a different dominant BBB.

## 3. Phytochemicals as Neurotherapeutics: Classes, Liabilities, Mechanisms of Actions

### 3.1. Polyphenols (Resveratrol, Quercetin, and Curcumin)

Polyphenols, particularly resveratrol, quercetin, and curcumin, represent the most extensively studied class of neuroprotective phytochemicals, attracting attention due to their pleiotropic activities and broad preclinical support in models of neurodegenerative disease [94,95,96,97,98,99,100]. Their neurotherapeutic potential rests on a complex repertoire of mechanisms that extend beyond simple radical scavenging [97,98,100,101]. Resveratrol activates SIRT1 signaling, promoting mitochondrial biogenesis and synaptic resilience, while quercetin modulates AMPK and Nrf2 pathways to counter oxidative stress and restore redox balance [99,101,102,103]. Curcumin has been shown to suppress NF-κB and Toll-like receptor signaling, thereby dampening neuroinflammatory cascades and protecting neuronal networks [35,70,98,101]. Collectively, these pathways converge to enhance neuronal survival, preserve cognitive function, and mitigate disease-associated cellular stress [94,98,99,100].

Despite these appealing mechanisms, polyphenols suffer from profound pharmacokinetic limitations [104,105,106,107]. All three undergo extensive first-pass metabolism through uridine 5′-diphospho-glucuronosyltransferase (UGT) and sulfotransferase (SULT) pathways, producing conjugated metabolites with limited biological activity [97,106,107,108]. The predominance of glucuronides and sulfates in circulation sharply reduces the availability of free aglycones that are more pharmacologically active [97,104,106,107,108]. Moreover, oral bioavailability is poor, with plasma concentrations of parent compounds often remaining below therapeutic thresholds even at high dietary intake [104,105,106,109]. Such metabolic liabilities have fueled a parallel interest in prodrug approaches and nanoformulations aimed at preserving active moieties for CNS delivery [98,100,105,110].

Even when absorbed, polyphenol penetration into the brain is further constrained by efflux transporters at the BBB [99,100]. P-gp and breast cancer resistance protein (BCRP) actively restrict their accumulation in brain parenchyma, while a “permeability paradox” emerges from the discrepancy between promising in vitro BBB transport studies and the much lower exposures seen in vivo [37,99,100,111]. This discordance reflects not only transporter activity but also systemic metabolism and protein binding, which together limit CNS bioavailability [100,104,105,106,107]. A more objective way to quantify formulation gain is to compare paired brain exposure metrics for the same payload administered as free compound versus nanoformulation. When dose, route, and sampling windows are matched, brain Cmax and brain AUC provide a direct readout of delivery advantage, while K_p,brain_ and K_p,uu,brain_ help separate true BBB transport from plasma driven carryover [112]. We therefore emphasize that claims of improved brain penetration should, where possible, be anchored to these paired pharmacokinetic comparisons rather than inferred from behavioral efficacy alone.

Nevertheless, polyphenols continue to serve as reference scaffolds in neurotherapeutic research, inspiring innovative strategies to overcome BBB constraints while maintaining their broad pharmacodynamic advantages [98,99,100,105,110].

### 3.2. Alkaloids (Berberine and Galantamine)

Alkaloids represent a chemically diverse class of CNS–active molecules with deep roots in both ethnopharmacology and modern clinical medicine [113,114]. Among them, berberine has emerged as a compelling yet pharmacokinetically problematic candidate [115,116]. It interacts strongly with organic cation transporters and is a recognized substrate of P-gp, factors that severely limit its absorption and systemic distribution [115,116,117]. Berberine undergoes rapid first-pass metabolism, exhibits low oral bioavailability, and suffers from pronounced metabolic instability, resulting in extremely poor CNS exposure despite promising neuroprotective and anti-inflammatory effects demonstrated in cellular and animal models [115,116,118]. These challenges have spurred interest in nanoparticle formulations and prodrug strategies designed to bypass efflux transport and enhance brain uptake [118,119,120,121,122,123].

By contrast, galantamine provides an example of a plant-derived alkaloid that has successfully transitioned into clinical practice as an approved therapy for Alzheimer’s disease [113,114,124,125]. Acting as a selective acetylcholinesterase inhibitor, it improves cholinergic transmission and demonstrates measurable cognitive benefits [114,124,125]. Interestingly, its central activity is not strictly proportional to plasma exposure, as galantamine crosses the BBB primarily through passive diffusion with potential contributions from carrier-mediated processes [70,126,127,128]. This selective permeability enables therapeutic CNS engagement even at moderate systemic concentrations, underscoring the importance of pharmacodynamic targeting in addition to pharmacokinetics [70,126,127].

Together, berberine and galantamine exemplify the so-called exposure–signal paradox at the BBB, where strong CNS effects can be achieved despite restricted or unpredictable drug penetration [70,126,127,128].

### 3.3. Terpenoids and Cannabinoids (Cannabidiol (CBD)/Δ9-Tetrahydrocannabinol (THC), Ginkgolides)

Lipophilic terpenoids and cannabinoids such as cannabidiol (CBD), Δ9-tetrahydrocannabinol (THC), and ginkgolides display paradoxical behavior at the BBB, where their high hydrophobicity does not consistently translate into effective CNS delivery [129,130,131]. CBD and THC are both highly lipophilic molecules, yet their brain penetration is actively curtailed by efflux pumps including P-gp and BCRP, which lower their effective concentrations in neural tissue [131,132,133]. Despite these restrictions, clinical and preclinical evidence demonstrates robust antiepileptic, anxiolytic, and analgesic activity, leading to regulatory approval of CBD for severe childhood epilepsies and THC formulations for spasticity and pain management in multiple sclerosis [134,135,136,137]. Their mechanisms are diverse, encompassing CB1 and CB2 receptor modulation, serotonergic signaling through 5-HT1A receptors, and anti-inflammatory as well as antioxidant actions, though their oral bioavailability remains low and interindividual variability in CNS exposure is considerable [133,138,139,140].

Ginkgolides, diterpenoid lactones derived from Ginkgo biloba, present a different profile, achieving moderate penetration into the CNS. Their primary mechanism of action involves antagonism of platelet-activating factor, a pathway linked to neuroinflammation and ischemic injury [141,142]. Preclinical findings suggest neuroprotective and anti-inflammatory potential, yet clinical trials have yielded mixed results, with benefits often modest and outcomes limited by poor BBB permeability and variable bioavailability [141,142]. The discrepancy between mechanistic promise and inconsistent clinical performance reflects the difficulty of translating terpenoid pharmacology into effective CNS therapeutics.

Together, cannabinoids and ginkgolides illustrate the so-called lipophilicity trap, in which excessive hydrophobicity, combined with efflux and metabolic instability, can paradoxically hinder brain delivery rather than facilitate it [129,130,131,132]. This paradox underscores the need for nuanced drug design and advanced delivery systems when considering terpenoids as neurotherapeutic candidates [139,140,143].

### 3.4. Formulation-Relevant Liabilities and Structure–Activity Relationship (SAR) Flags

Beyond class-specific mechanisms, the physicochemical properties of phytochemicals largely dictate their ability to cross the BBB and achieve therapeutic relevance [70,144,145]. Optimal penetration is typically observed in compounds with a logD between 1 and 3, balanced pK_a values that minimize ionization at physiological pH, a hydrogen bond donor count of two or fewer, and a hydrogen bond acceptor count not exceeding five [144,146,147]. Molecular flexibility is equally important, with fewer than ten rotatable bonds generally favoring permeability and sustained CNS exposure [144,148]. These criteria extend Lipinski’s Rule of Five into the realm of CNS drug-likeness and provide practical benchmarks for evaluating natural products [144,146,147].

Conversely, several red flags frequently emerge among phytochemicals. Excessive polarity or a topological polar surface area greater than 90 Å^2^ strongly predicts poor CNS penetration [144,146,148]. Similarly, a high density of hydrogen bond donors, often in the form of phenolic hydroxyl groups, correlates with both poor permeability and metabolic vulnerability through glucuronidation or sulfation [70,144]. These metabolic soft spots, common in polyphenols and terpenoids, reduce bioavailability and amplify efflux transporter recognition [149,150,151].

Early recognition of these liabilities through structure–activity relationship analysis and computational screening is therefore essential [144,152,153]. Such insights can guide the rational design of prodrugs and nanoformulations, improving bioavailability and transforming suboptimal scaffolds into viable neurotherapeutic candidates [4,145,154] (Table 2).

## 4. Nanomedicine Platforms for Blood–Brain Barrier (BBB) Delivery

This section surveys the main nanomedicine platform families used to move neuroactive payloads across the BBB and into brain tissue. We start with polymeric nanoparticles, since PLGA class systems set the translational baseline and illustrate how surface design and intranasal strategies can shift exposure. We then cover lipid carriers, from liposomes to SLNs, NLCs, and nanoemulsions, highlighting the tradeoffs between versatility, stability, and reproducibility. Next, we summarize dendrimers and micelles as programmable and responsive options for hard-to-deliver cargos. We close with inorganic, hybrid, and corona-controlled designs that add imaging, external control, or multifunctionality, but raise higher CMC demands.

### 4.1. Polymeric Nanoparticles (Poly(Lactic-Co-Glycolic Acid) (PLGA), Polyethylene Glycol (PEG)-PLGA, and Chitosan)

Polymeric nanoparticles have emerged as highly versatile carriers for CNS delivery, with poly(lactic-co-glycolic acid) (PLGA) considered the gold standard due to its biocompatibility, biodegradability, and track record of clinical translation [162,163,164]. Poly(butyl cyanoacrylate) (PBCA) nanoparticles are also among the longest-studied BBB nanocarriers and are frequently discussed in translational contexts because they offer a well-characterized preclinical brain delivery history [165,166,167]. The incorporation of polyethylene glycol (PEG) into PLGA scaffolds provides “stealth” properties by shielding the carrier from opsonization and prolonging its circulation half-life, thereby substantially enhancing the probability of crossing the BBB [168,169,170]. Beyond their pharmacokinetic advantages, PLGA and PEG-PLGA matrices are particularly well suited for encapsulating hydrophobic phytocompounds, thereby improving aqueous solubility and enabling sustained release profiles that minimize burst effects while maintaining therapeutic concentrations within neural tissue [163,169,171]. Several studies have highlighted that surface modifications, ranging from peptide ligands such as Angiopep-2 to functional protein corona interactions, can further refine nanoparticle selectivity for BBB transport and neuronal uptake [38,172,173,174].

Chitosan-based systems represent a complementary and increasingly significant strategy, exploiting their intrinsic cationic nature and mucoadhesive capacity [168,175]. When used either as a surface coating or as a hybrid scaffold with PLGA, chitosan enables tight interaction with mucosal surfaces and facilitates paracellular transport [168,175,176]. This property is particularly advantageous for intranasal administration, as demonstrated by formulations where PLGA nanoparticles embedded within chitosan microparticles achieved enhanced uptake across the olfactory mucosa and direct delivery to the brain [168,176,177]. Intranasal chitosan–PLGA carriers have been successfully applied to deliver repurposed chemotherapeutics such as gemcitabine for glioblastoma therapy, achieving tumor-selective release while bypassing systemic clearance [164,168,174].

Quantitative pharmacokinetic assessments underscore the translational potential of these approaches [162,170,176]. Enrichment analyses of brain-to-plasma distribution indicate that optimized PLGA- and chitosan-based delivery systems can increase cerebral accumulation by several fold relative to unformulated compounds. These findings support the use of polymeric nanomedicine not merely as a means to enhance phytocompound bioavailability, but as a deliberate strategy to harness transport mechanisms for targeted CNS therapy [163,169,170,176].

### 4.2. Lipid Carriers (Liposomes, Solid Lipid Nanoparticles (SLNs), and Nanoemulsions)

Lipid-based nanocarriers have become central to brain-targeted delivery, with liposomes representing the archetypal bilayer system [178,179,180,181]. Some liposomal formulations and extracellular vesicle-based systems have already advanced into clinical trials, and selected lipid carriers are in clinical use for CNS relevant indications, supporting their translational maturity [181,182] Their amphiphilic structure allows for simultaneous encapsulation of hydrophilic and hydrophobic compounds, while PEGylated liposomes confer stealth properties that prolong circulation time and enhance BBB penetration [178,183,184]. This versatility has been leveraged in multiple preclinical models, where PEGylated formulations not only improved stability but also demonstrated controlled biodistribution within brain parenchyma [178,183,185]. The bilayer’s modularity also facilitates functionalization with targeting ligands, thereby adding a level of precision that polymeric systems often struggle to replicate [178,185].

Solid lipid nanoparticles (SLNs) and nanostructured lipid carriers (NLCs) have advanced the field by addressing limitations of traditional liposomes, particularly with respect to stability and drug-loading capacity [161,178,186]. SLNs, composed of solid lipids at body temperature, offer biocompatibility and controlled release but are often restricted by lower payload efficiency [161,187]. In contrast, NLCs incorporate both solid and liquid lipids into their matrix, thereby providing greater drug accommodation and reducing the risk of expulsion during storage [157,161,188]. Comparative studies consistently highlight the superior stability and performance of NLCs over SLNs, particularly for long-term formulations aimed at chronic neurodegenerative diseases [157,188,189].

Nanoemulsions extend the potential of lipid systems by enabling rapid and direct intranasal delivery to the brain [188,190]. Their small droplet size promotes fast absorption through the olfactory epithelium, a pathway particularly attractive for bypassing systemic metabolism [188,190,191]. However, reproducibility in manufacturing, along with challenges in preventing aggregation and ensuring shelf-life stability, remains an unresolved hurdle [190,192,193]. As recent in vivo studies emphasize, the promise of nanoemulsions lies in their high uptake efficiency, yet their clinical translation will depend on improved standardization and stabilization strategies [188,190,193].

### 4.3. Dendrimers and Micelles

Dendrimers represent one of the most structurally sophisticated nanocarriers for CNS therapy [194,195]. Their branched, tree-like architecture provides internal cavities for drug encapsulation and a multivalent surface for ligand attachment, making them particularly effective in tuning brain-specific targeting [194,195,196]. The critical challenge, however, lies in balancing ligand density: while higher densities can enhance receptor-mediated transcytosis, excessive functionalization often increases steric hindrance or cytotoxicity [194,196,197]. Studies with polyamidoamine (PAMAM) and carbosilane dendrimers demonstrate that size and surface charge strongly dictate BBB penetration, with mixed-surface or PEGylated variants offering improved biocompatibility and reduced clearance [198,199,200,201]. Such design nuances highlight the delicate trade-off between maximizing efficacy and minimizing off-target toxicity in vivo [194,196,199].

Micelles, by contrast, exploit their amphiphilic organization to solubilize poorly water-soluble compounds, a feature particularly advantageous for phytochemicals and nucleic acids [202,203]. Their self-assembly into nanosized aggregates enables responsiveness to physiological triggers, such as acidic pH or enzyme activity, resulting in controlled drug release within the brain microenvironment [204,205,206]. Optimization of micelle size and zeta potential has been shown to extend circulation while facilitating BBB passage without rapid renal clearance [198,202,204]. Recent developments in cation-free micelles for siRNA delivery illustrate how fine-tuned charge control can reduce cytotoxicity while preserving efficient uptake in glioblastoma models [202,207]. Together, dendrimers and micelles provide complementary strategies: dendrimers excel at multivalent, targeted interactions, while micelles offer dynamic, responsive platforms for solubilization and triggered release [198,203].

### 4.4. Inorganic/Carbon Nanostructures

Carbon- and inorganic-based nanostructures have carved a distinct niche in BBB research due to their dual roles as carriers and imaging agents [208,209,210]. Gold nanomaterials, including AuNPs, have also been reported to cross the BBB in multiple preclinical models, and selected gold-based systems have progressed toward translational evaluation, including early clinical trial activity [4,211,212]. Carbon dots and quantum dots, for example, exhibit intrinsic fluorescence, enabling simultaneous drug delivery and real-time tracking [208,213,214,215]. This built-in diagnostic capacity underpins their promise for theranostic applications, particularly in neurodegenerative disease models where monitoring biodistribution is critical [208,216,217,218]. Magnetic nanoparticles extend this paradigm further by enabling magnetically guided delivery, offering external control over accumulation in targeted brain regions [166,181,210,217,219,220]. Such approaches not only enhance precision but also reduce systemic exposure, positioning these nanostructures as powerful candidates for next-generation CNS therapeutics [209,210,217].

Despite these advantages, their translation faces significant barriers [213,221]. The long-term biocompatibility of quantum dots and carbon nanodots remains uncertain, with concerns over oxidative stress, protein corona formation, and potential accumulation in neural tissues [208,213,221,222]. Magnetic nanoparticles, while effective in guidance and imaging, also raise questions about clearance and toxicity with repeated use [210,217,219,221]. Animal studies have demonstrated promising biodistribution patterns, yet discrepancies in chronic safety outcomes highlight the need for rigorous toxicological evaluation before clinical adoption [213,221,223,224]. In this context, the field is actively exploring polymer-coated and functionalized variants to mitigate oxidative damage while preserving the diagnostic and therapeutic potential [210,217,219,221]. Together, inorganic and carbon nanostructures embody both the allure and caution of theranostic nanomedicine: they provide unparalleled control and visibility but demand equally careful assessment of their long-term biological footprint [209,213,221].

### 4.5. Hybrid/Biodegradable and Protein Corona Control

Hybrid nanomedicine platforms are increasingly recognized as promising strategies for crossing the BBB because they combine complementary features of polymeric, lipid, and inorganic scaffolds [72,225,226]. Polymeric–lipid hybrids, for example, integrate the structural stability of polymers with the biocompatibility and drug-loading flexibility of lipid layers, while inorganic–organic hybrids offer imaging capabilities alongside controlled drug release [72,227,228]. A recurring theme in these designs is the fine-tuning of surface charge; adjusting zeta potential can minimize opsonization and prolong circulation, yet excessive neutralization may compromise cellular uptake [72,155,229]. Dual-targeting systems, such as polyanionic polymalic acid nanodrugs conjugated with Angiopep-2, illustrate how surface chemistry can be leveraged to maintain stability while enabling efficient receptor-mediated transport into the brain [229,230,231].

A critical determinant of in vivo performance lies in protein corona formation, which reshapes nanoparticle identity immediately upon systemic entry [232]. Far from being an inert byproduct, the corona can hinder transcytosis, reduce tumor selectivity, or alternatively, be engineered to guide delivery [232]. Strategies to regulate this interface include pre-coating with tunable surfactants, exploiting biomimetic exosome-mimetic shells, or even deliberately co-opting serum proteins to enhance stealth [232]. Such approaches demonstrate that corona engineering is not merely defensive but can be actively harnessed to improve BBB passage and targeting precision.

At the same time, clinical translation of these platforms hinges on biodegradability and safety [72,155,225]. Polyanhydride-based carriers and bioinspired protein–polymer nanocapsules exemplify progress toward fully degradable designs, yet challenges remain regarding reproducibility, long-term safety, and scale-up [72,155]. As recent reviews emphasize, the success of hybrid systems will depend not only on their multifunctional design but also on overcoming regulatory hurdles by proving that corona control and biodegradability can coexist without compromising efficacy [72,225,226].

### 4.6. Literature Snapshot

Comparative evaluations of nanomedicine platforms highlight how material choice shapes brain delivery outcomes [66,233,234]. Head-to-head studies reveal that PLGA nanoparticles carrying flavonoids often outperform liposomes in terms of controlled release and systemic stability, while liposomes enable faster brain penetration but can be prone to leakage and reduced retention [235,236,237]. Similarly, dendrimers and micelles demonstrate complementary advantages when loaded with peptide cargos: dendrimers benefit from multivalency that enhances receptor-mediated uptake, whereas micelles provide stimulus-responsive release and better solubilization of hydrophobic payloads [238,239,240]. Endpoints such as BBB permeability ratios, neurobehavioral outcomes in stroke or neurodegeneration models, and toxicity remain the unifying benchmarks across these comparisons [66,233,234].

The collective evidence makes one principle clear: there is no universal best nanomedicine platform [233,234,237]. The optimal choice is highly context-driven, defined by the therapeutic payload, disease model, and the balance between efficacy and safety [66,233,234]. Surface chemistry and biological targeting often make or break brain delivery—hence a focused look at ligands, valency, and stimuli [236,238,239] (Table 3).

## 5. Targeting and Stimuli Strategies

This section focuses on the control knobs that turn a generic nanocarrier into a brain-directed system. We first cover receptor-mediated transcytosis ligands, since TfR, LRP1, IR, and related pathways remain the most rational way to cross intact endothelium. We then discuss adsorptive and CPP strategies, which can drive strong uptake but need smarter selectivity to stay safe. Next comes multivalent and dual targeting, where BBB entry and disease homing are combined. Finally, we summarize stimuli-responsive designs that time and localize release using internal cues or external triggers.

### 5.1. Receptor-Mediated Transcytosis (RMT) Ligands

Receptor-mediated transcytosis (RMT) has emerged as the cornerstone of BBB targeting, providing a rational route for therapeutic entry into the CNS [77]. Among the most extensively characterized receptors, the transferrin receptor (TfR), low-density lipoprotein receptor-related protein 1 (LRP1), insulin receptor (IR), and LDL receptor (LDLR) have each been exploited to shuttle biologics, peptides, and nanocarriers across endothelial cells [63,251]. Ligands such as Angiopep-2, apolipoprotein E (ApoE)-mimetics, and engineered transferrin derivatives illustrate how endogenous trafficking machinery can be co-opted without major structural perturbations to the BBB [63,251]. This strategy has been validated in multiple preclinical models and increasingly in human-relevant systems, including iPSC-derived BBB platforms that closely recapitulate receptor dynamics [252,253].

The success of RMT hinges on a finely tuned balance between ligand affinity and avidity [77,254]. Excessively strong binding may lead to receptor saturation or sequestration in lysosomes, while weak interactions risk premature dissociation and suboptimal delivery [75,254]. Mathematical modeling and linker engineering have refined our understanding of these trade-offs, highlighting an “affinity window” that permits recycling and productive transcytosis [254,255]. Yet, competition with endogenous ligands imposes a physiological ceiling effect, particularly for receptors like TfR and IR that are critical for nutrient homeostasis [255,256]. This ceiling necessitates dosing strategies and ligand modifications that preserve BBB transport without displacing natural substrates.

Translational progress has been most visible in the diversification of ligand classes. Antibody fragments and bispecific constructs with optimized linkers now outperform earlier monoclonals in preclinical BBB assays [37,257,258]. In parallel, short peptides, nanobodies, and aptamers provide modular alternatives that reduce immunogenicity while retaining high receptor specificity [75,259,260]. Collectively, these innovations illustrate how the conceptual framework of RMT is being transformed into clinically viable neurotherapeutic strategies, setting the stage for next-generation delivery systems tailored to the diseased brain microenvironment.

### 5.2. Adsorptive and Cell-Penetrating Peptide (CPP) Strategies

Adsorptive-mediated transcytosis and cell-penetrating peptides (CPPs) represent alternative strategies to receptor-based delivery for crossing the BBB [76,261,262]. Their principle rests on cationic surfaces that engage in electrostatic interactions with the negatively charged glycocalyx of endothelial cells, thereby initiating uptake [261,263]. Classic CPPs such as TAT, penetratin, and rabies virus glycoprotein (RVG) have been widely studied and consistently demonstrate high internalization efficiency across a range of in vitro and in vivo models [262,264,265]. This strong uptake capacity has made them attractive tools for brain-directed delivery of proteins, nucleic acids, and nanocarriers [261,262,265]. However, the very same non-specificity that enables broad penetration also increases the risk of cytotoxicity and off-target accumulation in peripheral tissues, posing major challenges for clinical translation [266,267,268].

To overcome these limitations, newer designs exploit reversible or conditional activation of cationic charges [269,270]. Charge-switchable coatings that remain neutral in circulation but expose CPP activity in acidic or enzymatically active microenvironments offer a way to enhance specificity while reducing systemic toxicity [269,270]. Stimulus-responsive CPPs integrated into nanogels, or liposomes can be selectively unveiled in glioma or inflamed brain regions, thereby combining the high uptake efficiency of adsorptive strategies with a more targeted therapeutic profile [269,271,272]. These innovations suggest that adsorptive and CPP approaches, once criticized for their lack of selectivity, may reemerge as valuable complements to receptor-mediated systems when combined with smart design principles [262,266,269].

### 5.3. Multivalent/Dual-Targeting Designs

Multivalent and dual-targeting strategies build on the idea that no single ligand can fully capture the complexity of BBB transport and disease-specific recognition [273,274,275]. By combining receptor-mediated transcytosis ligands with disease-associated epitopes, such as amyloid-binding motifs in Alzheimer’s disease or glioma-homing peptides, researchers aim to achieve both efficient barrier penetration and selective accumulation in pathological tissue [256,274,276]. This layered approach provides synergy, since one ligand optimizes endothelial uptake while the second guides delivery toward neuronal or tumor targets [276,277,278]. The result is not simply additive transport but enhanced fidelity of targeting, often translating into improved therapeutic outcomes in preclinical models [276,277,279].

Nonetheless, designing dual-decorated nanocarriers requires careful calibration [275,279,280]. Steric hindrance between ligands, suboptimal linker lengths, or excessive surface density can compromise binding efficiency and even trigger accelerated clearance [279,280]. Several studies illustrate both promise and pitfalls [274,276,277]. In Alzheimer’s disease models, nanoparticles bearing transferrin and neuron-targeting Tet1 peptides showed superior cognitive rescue compared with single-ligand systems [274,276]. In glioblastoma, lipid nanocarriers co-functionalized with Angiopep-2 and trans-activator of transcription (TAT)-achieved deeper tumor penetration and survival benefits [274,276]. These examples highlight how multivalent strategies, when optimized, can balance BBB entry with precision delivery, positioning them as one of the most forward-looking directions in neurotherapeutics [37,75,273].

### 5.4. Stimuli-Responsive Systems

Stimuli-responsive systems harness both endogenous and exogenous cues to achieve precise control over drug delivery across the BBB [38,281,282]. Internal triggers such as acidic pH gradients, redox imbalances, and overexpressed enzymes in the tumor microenvironment have been successfully integrated into nanocarriers to enable controlled and site-specific release [281,283,284]. pH-sensitive polymers, disulfide-cleavable linkers, and enzyme-activated coatings exemplify this strategy, ensuring that therapeutic cargo remains stable in circulation yet becomes rapidly available once inside diseased brain regions [198,284,285]. These approaches not only enhance local efficacy but also reduce systemic exposure, thereby addressing one of the central challenges of neurotherapeutics [235,282].

**Figure 2:**
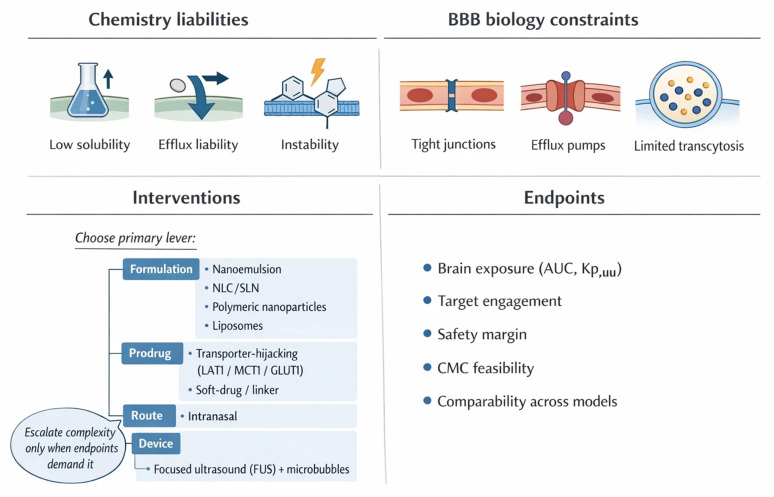
Decision tree for selecting CNS delivery strategies by payload liabilities and clinical context. The schematic provides a pragmatic decision tree to match a CNS payload to the most efficient delivery strategy. The workflow begins with dominant liabilities: (i) solubility/dissolution limitation, (ii) efflux liability (e.g., P-gp/BCRP/MRPs), and (iii) chemical or metabolic instability. Solubility-driven failure routes first to formulation solutions (solubilizing vehicles, lipid systems, polymeric nanoparticles, mucoadhesive/intranasal formats when appropriate). Predominant efflux liability routes to prodrug design (efflux-evading promoieties or transporter-hijacking approaches such as GLUT1/LAT1/MCT1) and/or carrier shielding/targeting (stealth/corona control; RMT ligands such as TfR/LRP1). Instability-driven failure routes to protective encapsulation or stability-optimized prodrugs with controlled release. Clinical context modifiers (need for rapid onset, diffuse vs. focal pathology, and tolerability constraints) direct selection of route (e.g., intranasal bypass) or focused ultrasound (FUS) + microbubbles for localized BBB modulation. Each terminal node specifies decision-grade endpoints: brain exposure (AUC, K_p,brain_, K_p,uu_), target engagement, safety margin, and CMC scalability. AUC, area under the curve; BBB, blood–brain barrier; BCRP, breast cancer resistance protein; CMC, chemistry, manufacturing, and controls; CNS, central nervous system; FUS, focused ultrasound; GLUT1, glucose transporter 1; K_p,brain_, brain-to-plasma partition coefficient; K_p,uu_, unbound brain-to-plasma partition coefficient; LAT1, L-type amino acid transporter 1; MCT1, monocarboxylate transporter 1; MRPs, multidrug resistance-associated proteins; P-gp, P-glycoprotein; RMT, receptor-mediated transcytosis; TfR, transferrin receptor; LRP1, low-density lipoprotein receptor-related protein 1.

External stimuli offer an additional dimension of spatiotemporal precision [235,282,283]. Magnetic fields, focused ultrasound, and light-based activation provide reversible and non-invasive triggers that can be synchronized with drug administration [282,286,287]. Such methods have been paired with polymeric and lipid nanocarriers to achieve on-demand release and deep penetration into glioblastoma tissue [4,282,287]. However, questions of safety, reproducibility, and clinical feasibility remain unresolved, particularly for modalities requiring specialized equipment or prolonged exposure [235,288,289]. Balancing innovation with practicality is crucial as these systems move [235] toward translation [235,281,290]. Beyond targeting, alternative routes and device-enabled openings can bypass or transiently relax the barrier [4,59] (Table 4, Figure 2).

## 6. Alternative Routes and Device-Enabled Blood–Brain Barrier (BBB) Opening

This section covers delivery options that sidestep the usual BBB rules or briefly relax them on demand. We start with intranasal nose to brain delivery, since it can bypass first pass metabolism and pair naturally with mucoadhesive gels and nanoformulations. We then discuss focused ultrasound plus microbubbles, a switch like method that opens the BBB locally and reversibly with high spatial precision. Finally, we summarize older chemical and osmotic methods and convection-enhanced delivery, emphasizing where they still fit and where risk or practicality limits translation.

### 6.1. Intranasal Nose-to-Brain

The intranasal route exploits the unique anatomical connectivity between the nasal cavity and the brain through the olfactory epithelium and the branches of the trigeminal nerve [188,302]. These pathways enable both intra- and extra-neuronal transport, providing rapid and direct access to the CNS while bypassing systemic circulation and hepatic first-pass metabolism [188,303]. Such direct trafficking has been demonstrated for a wide range of small molecules, peptides, and nanocarrier systems, reinforcing the potential of this route for delivering neuroprotective phytochemicals and engineered prodrugs [188,304,305].

Formulation science has been central to enhancing this delivery mode [302,304]. Mucoadhesive in situ gels, often thermo- or ion-responsive, prolong nasal residence time and counteract mucociliary clearance, while nanoemulsions improve solubility and stability of hydrophobic polyphenols such as curcumin or resveratrol [188,306,307]. Nanoparticulate systems—ranging from lipid-based carriers to chitosan-modified polymeric nanoparticles—further allow surface functionalization for improved permeability and targeted release [155,305,308]. Prodrug strategies that exploit enzymatic conversion within the nasal mucosa are being increasingly explored to improve bioavailability and sustain brain exposure [302].

Despite this promise, translational hurdles remain [304,309]. Anatomical variability, short retention time, and interindividual differences in nasal airflow complicate dosing precision and reproducibility in humans [188,309]. Ergonomic device design, accurate metered dosing, and integration with pharmacokinetic modeling will be essential for clinical translation [188,309]. Large-scale human trials, coupled with regulatory harmonization, are still required before intranasal nanoformulations can be considered reliable delivery systems for neurotherapeutics [302,304,310].

### 6.2. Focused Ultrasound (FUS) + Microbubbles

Focused ultrasound combined with circulating microbubbles has emerged as one of the most precise approaches to transiently opening the BBB [311,312]. The mechanism relies on acoustic cavitation, in which microbubbles oscillate in response to ultrasound exposure, producing localized shear stress on the vascular endothelium [311,313,314]. This process induces mechanoporation and loosening of tight junctions, thereby increasing paracellular permeability in a controlled and reversible manner [311,313,314]. Importantly, both stable and inertial cavitation contribute to permeability enhancement, yet parameters must be carefully tuned to avoid endothelial damage or hemorrhage [313,315,316]. The reversible nature of the opening distinguishes FUS from chemical osmotic methods, as the barrier typically restores within hours [311].

A major strength of this technology lies in its spatiotemporal precision [311,317,318]. MRI-guided or neuronavigation-based systems allow targeting of submillimeter brain regions, enabling localized drug accumulation with minimal off-target exposure [317,319,320]. Real-time cavitation monitoring, coupled with feedback-controlled ultrasound delivery, provides essential safety guardrails, reducing risks of edema, neuroinflammation, or microvascular injury [315,321,322]. Longitudinal studies in both primates and humans confirm the feasibility of repeated sessions without significant adverse cognitive effects, although vigilance for subtle inflammatory responses remains necessary [313,323,324].

**Figure 3:**
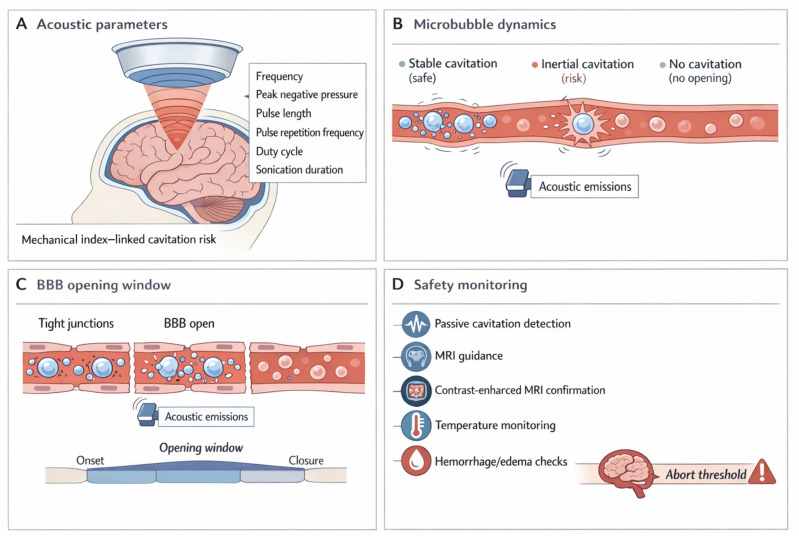
Focused ultrasound (FUS) delivery paradigm: acoustic control, microbubble cavitation, transient blood–brain barrier (BBB) opening, and safety monitoring. The schematic summarizes the focused ultrasound (FUS)–microbubble paradigm for transient, spatially targeted BBB modulation to enable CNS delivery. (**A**) Acoustic parameters (frequency, peak negative pressure, pulse length, pulse repetition frequency, duty cycle, and sonication duration) set the cavitation regime and therefore the balance between efficacy and risk. (**B**) Microbubble dynamics span a spectrum from no response (insufficient opening) to stable cavitation (desired oscillation that increases permeability) and inertial cavitation (collapse associated with vascular damage risk). These regimes are inferred in real time using acoustic emissions and passive cavitation detection to support parameter adjustment and stop rules. (**C**) BBB opening window is depicted as a reversible permeability increase (minutes–hours) during which small molecules, prodrugs, and nanocarriers can cross the endothelium; closure restores barrier integrity. (**D**) Safety monitoring includes image-guided targeting (MRI), confirmation of opening (contrast-enhanced MRI), surveillance for edema or microhemorrhage, and temperature/physiological checks where relevant. Together, the framework links controllable acoustic inputs to microbubble behavior, delivery timing, and verification steps required for reproducible, trial-grade BBB opening. BBB, blood–brain barrier; CNS, central nervous system; FUS, focused ultrasound; MRI, magnetic resonance imaging.

Clinical translation is already well under way [317]. Phase I trials in gliomas, Alzheimer’s disease, and Parkinson’s dementia consistently report tolerability and transient BBB disruption, with imaging confirming enhanced delivery of chemotherapeutics, antibodies, and nanoparticles [313,317,325]. While no trials have yet evaluated phytochemicals or natural prodrugs directly, the compatibility of FUS with nanocarriers and controlled-release systems makes such applications plausible [312,314,325]. Integrating polyphenol-based therapeutics into FUS platforms could represent a novel frontier for noninvasive neuroprotection and disease modification [314,325] (Figure 3).

### 6.3. Chemical/Osmotic Opening and Convection-Enhanced Delivery (CED)

Classical BBB-disruption strategies such as intra-arterial mannitol, DMSO co-solvent effects, and bradykinin analogs can transiently loosen tight junctions, yet their clinical utility has waned [288,326]. The reasons are consistent across reviews: non-selective permeability increases, variable magnitude and duration of opening, and procedure-related risks, including seizures, edema, or ischemic events [327,328]. Lack of spatiotemporal control and systemic toxicities further erode risk–benefit in longitudinal care [326]. For phytochemicals and natural prodrugs, which often require sustained or repeated exposure, these invasive and poorly tunable methods are a poor fit; cumulative toxicity and patient burden compound the translational gap [326,329].

CED preserves a niche when focal, high-dose deposition is essential, as in gliomas or diffuse midline lesions, particularly with implantable ports and image-guided catheters, despite technical complexity and heterogeneity of distribution [330,331,332]. When chemistry can carry the payload, prodrugs simplify the problem, if transporter hijacking and cleavage are tuned correctly [333,334] (Table 5). Combination nanomedicine is also gaining traction, especially when a single payload cannot cover the biology of neuroinflammation, oxidative stress, and circuit level dysfunction at once [335]. In practice, this means co-loading two phytochemicals, pairing a phytochemical with a peptide or nucleic acid, or integrating targeting with a triggerable release layer [336]. These fully functional systems are usually still preclinical, and they often trade elegance for manufacturability because every added function expands the CMC burden and increases batch sensitivity [337]. For short-term translation, the most credible combination strategy is modular; use clinically mature carriers or routes, then add one additional layer of control such as a ligand or a device-enabled BBB opening step, rather than stacking multiple innovations simultaneously [338].

## 7. Prodrugs and Transporter Hijacking

This section shifts from carriers to chemistry, focusing on prodrugs that use BBB transport rules to their advantage. We begin with transporter hijacking, especially LAT1, because it offers a direct, mechanistic route for small molecules to cross intact endothelium. We then cover lipidization and soft drug concepts, which tune passive diffusion and systemic clearance by design. Next, we separate solubility boosters from true BBB permeability modulators, since higher plasma exposure is not the same as higher brain exposure. Finally, we discuss nano prodrug conjugates that merge controlled release with prodrug activation, and we flag the CMC hurdles that still limit clinical uptake.

### 7.1. Large Neutral Amino Acid Transporter 1 (LAT1)-/Monocarboxylate Transporter 1 (MCT1)-/Glucose Transporter 1 (GLUT1)-Targeted Prodrugs

Among the influx transporters that shape small-molecule entry into the brain, the large neutral amino acid carrier LAT1 has emerged as the most exploited in prodrug design [346,347,348]. LAT1 is highly expressed on the luminal side of brain capillaries and recognizes aromatic and branched-chain amino acids as substrates [58,348,349]. By conjugating drugs with phenylalanine, tyrosine, or related promoieties, it is possible to achieve carrier-mediated uptake that circumvents passive BBB limitations [346,347,350]. LAT1-linked prodrugs of valproic acid, ferulic acid, and NSAIDs have shown superior brain penetration, and the Xiong 2021 dataset provides compelling evidence that conjugated neurotherapeutics not only cross the BBB but also accumulate within neurons, astrocytes, and microglia, confirming cellular specificity of uptake [351,352,353].

Kynurenine-inspired prodrugs offer a pragmatic way to turn kynurenic acid (KYNA)-like polarity from a liability into a controllable design variable. Compounds such as 4-chlorokynurenine temporarily mask polar functionality via halogenation, improving systemic handling and, potentially, CNS exposure [354,355]. After conversion, active kynurenic acid analogs can emerge, including 7-chlorokynurenic acid, which targets N-methyl-D-aspartate (NMDA) receptors by blocking the glycine site and thereby constraining excitotoxic drive [356,357]. Small structural edits can have outsized effects [354,355]. Translationally, 4-chlorokynurenine showed limited antidepressant efficacy in Phase II treatment-resistant depression, yet it continues to be explored across neurological and pain indications, alongside higher potency derivatives such as 4,6-dichlorokynurenine [354,358,359].

Kynurenine analogs can function as prodrug-like refinements of the KYNA scaffold, where side-chain edits tune exposure first and pharmacology follows. The SZR series is a good example of SAR in action. SZR-72 adds a modest methyl group, yet it is associated with stronger neuroprotection, improved BBB penetration, and measurable behavioral modulation, hinting that small steric nudges can unlock CNS activity [111,360]. SZR-104 takes a more electronic approach, introducing a polar ring system at C3 that still delivers high BBB permeability and neuroprotection in sepsis models [361,362]. Other members diversify the profile. SZR-109 combines robust BBB entry with suppression of TNF-α, upregulation of TSG-6, and anticonvulsant effects [360,362]. Translation remains the bottleneck, as safety signals such as off-target kinase inhibition with SZR-105 demand careful optimization and smarter combinations, including pairing with IDO inhibitors to reshape pathway flux [363,364]. Overall, the SZR series represents promising exposure-optimized KYNA analogs with multifunctional neuroprotective and anti-inflammatory effects but requires further development to address safety and translational hurdles.

Beyond LAT1, other solute carriers are beginning to attract attention [61,348]. Monocarboxylate transporter 1 (MCT1) recognizes lactate and pyruvate analogues, providing a scaffold for monocarboxylate-linked prodrugs, whereas GLUT1, the primary glucose transporter, can be hijacked via glucose conjugation [58,61,348]. Proof-of-principle studies demonstrate that indomethacin and ketoprofen conjugated to glucose traverse the BBB in rodents, though kinetic competition with endogenous glucose poses significant challenges [58,353]. These strategies illustrate the expanding toolkit for tailoring prodrug chemistry to align with the substrate repertoire of BBB carriers [58,348].

Transporter hijacking, however, is not without risk [346,365]. Kinetic constraints such as K_m_ and V_MAX_ dictate the efficiency of uptake, and saturation by high-affinity endogenous substrates can diminish drug delivery [58,347]. Moreover, transporter expression varies across species, complicating preclinical-to-clinical translation [61,348]. LAT1 prodrugs are generally selective, off-target interactions and potential saturation effects remain critical safety considerations [351,366,367]. The challenge now lies in fine-tuning conjugate chemistry to balance affinity, stability, and enzymatic cleavability in the brain while minimizing systemic exposure [349,350,352].

### 7.2. Lipidization, Soft Drugs, Self-Immolative Linkers

Lipidization remains one of the oldest yet most versatile strategies for enhancing drug penetration into the brain [368,369,370]. By appending lipophilic chains or glyceride motifs, polar APIs can acquire sufficient passive diffusion across endothelial membranes, provided the modifications are designed for efficient cleavage once in the CNS [368,369,370]. This balance between increased lipophilicity and metabolic lability is critical: too stable and the parent drug may not be released; too labile and systemic hydrolysis prevents brain delivery [368,370]. The approach has been applied successfully to small neuroactive agents, though reproducibility across species remains a central design challenge [369,371].

In parallel, soft drug concepts introduce the inverse logic: compounds are deliberately engineered for predictable inactivation outside the CNS, ensuring that only a fraction escapes rapid metabolism and reaches the brain [368,370]. Self-immolative linkers add yet another layer of sophistication, exploiting pH gradients, enzyme expression, or redox triggers to launch controlled cleavage cascades [372,373,374]. Modern designs favor traceless release, often with dual stimuli or cascade amplification to achieve brain-first activation while avoiding premature systemic leakage [372,373,375]. The guiding rule across these platforms is to harmonize stability, trigger sensitivity, and cleavage kinetics so that release occurs only under CNS-relevant conditions, minimizing off-target toxicity while maximizing therapeutic gain [372,373,375].

### 7.3. Solubility Boosters (Cyclodextrins, Co-Crystals, Ion Pairing)

Cyclodextrins have been widely used to improve aqueous solubility through inclusion complexes that sequester hydrophobic moieties within their cyclic cavities [148,376,377]. This strategy can significantly enhance systemic exposure and oral bioavailability, yet it offers little direct benefit for BBB permeation, as the bulky complexes rarely cross endothelial tight junctions intact [376,378,379]. Their role is therefore supportive: enabling consistent systemic levels that may feed into other brain-targeted strategies rather than acting as genuine CNS delivery enhancers [377,380,381].

Co-crystals and ion pairing occupy a more dynamic niche [382]. Co-crystals modify dissolution rates and solubility without altering the pharmacodynamic profile of the parent drug, creating opportunities for predictable exposure kinetics [382,383,384]. Ion pairing, in contrast, transiently adjusts lipophilicity by associating ionizable drugs with counterions, thereby improving membrane partitioning and yielding short-lived permeability gains [385,386,387]. The central distinction is crucial: while all three approaches may improve systemic bioavailability, only certain ion-pairing strategies directly modulate BBB permeability [385,386,387]. Recognizing this separation between systemic solubility enhancers and true BBB permeability modulators is essential when positioning such methods within prodrug pipelines [386,387,388].

### 7.4. Nano–Prodrug Conjugates

Nano–prodrug conjugates represent a convergence of nanomedicine and classical prodrug chemistry [389,390,391]. In these systems, nanocarriers such as polymers, liposomes, or albumin-binding constructs are covalently linked to prodrug moieties, creating assemblies that combine carrier stability with controlled release [389,391,392]. Examples include polymer–drug conjugates that self-assemble into micelles or nanoparticles, and liposome–prodrug hybrids that integrate covalently modified drugs into bilayer structures [242,389,393]. Activation is then triggered by tumor- or CNS-relevant stimuli such as redox gradients, pH shifts, or enzyme cleavage, ensuring spatially restricted release [284,393,394].

The rationale for this complexity is strongest when dealing with drugs that have narrow therapeutic windows or poor solubility, where conventional formulations risk systemic toxicity or inadequate exposure [391,395]. By embedding prodrug chemistry within nanocarriers, it becomes possible to synchronize delivery, minimize premature release, and improve therapeutic indices [389,395,396]. Yet translation remains challenging [396,397,398]. Manufacturing reproducibility, batch-to-batch stability, and regulatory pathways for hybrid entities blur the lines between drug and device, complicating approvals [397,398,399]. Scalability and quality control of multifunctional prodrug nanocarriers are further hurdles that limit current clinical penetration despite compelling preclinical evidence [397,398,400]. Delivery vectors and prodrugs must be vetted in models that actually predict human exposure—next we align models with decision-grade endpoints [397,398] (Table 6).

## 8. Biogenic and Exosome-Mimetic Vesicles

### 8.1. Mammalian Exosomes

Mammalian exosomes have attracted intense interest as endogenous delivery vehicles, given their origin from neuronal, immune, and stem-cell lineages [418,419]. Neuron-derived vesicles display inherent neurotropism, while macrophage or dendritic cell exosomes often retain immunological signaling capabilities that can be leveraged for targeted delivery [419,420]. Stem cell-derived vesicles, particularly those from mesenchymal sources, exhibit regenerative properties and have been applied in models of neuroinflammation and tissue repair [421,422]. This natural diversity provides a menu of options for CNS-directed therapy, with the vesicle’s parent cell type influencing both tropism and therapeutic payload [421,423].

Several methods exist for incorporating cargo into exosomes [420,424]. Electroporation transiently disrupts vesicle membranes to load nucleic acids, while passive incubation exploits lipid bilayer partitioning [420,425]. Sonication and extrusion, though less subtle, can increase loading efficiency for small molecules and proteins [425,426]. More sophisticated approaches combine chemical conjugation or ligand decoration to engineer selective homing properties, extending beyond the vesicle’s innate targeting profile [424,427].

Despite these advantages, translational obstacles remain formidable [428,429]. Batch-to-batch variability complicates reproducibility, and large-scale production has yet to reach regulatory-grade consistency [418,428,429]. Issues of heterogeneity, yield, and purification standards pose barriers to clinical adoption, while classification of exosomes as biologics, devices, or drug–biologic hybrids remains unresolved [430,431]. Thus, mammalian exosomes stand at the intersection of promise and challenge, offering unmatched biocompatibility but demanding rigorous solutions in scalability and regulation before they can function as reliable neurotherapeutic vectors [418,419].

### 8.2. Plant-Derived Extracellular Vesicles

Plant-derived extracellular vesicles (PDEVs) are emerging as abundant, low-costnanocarriers harvested from edible sources such as ginger, grape, and citrus [432,433,434]. Their natural stability, low immunogenicity, and tolerance to gastrointestinal conditions make them particularly attractive for oral or intranasal administration, routes that remain challenging for mammalian exosomes [432,435,436]. PDEVs also carry intrinsic bioactive metabolites, adding antioxidant and anti-inflammatory potential to their delivery role [434,437,438].

Despite these advantages, several limitations temper enthusiasm [432,433]. Vesicle heterogeneity across plant species and even between batches complicates reproducibility, while the mechanisms by which PDEVs interact with or traverse the BBB remain poorly defined [432,438,439]. Preclinical studies demonstrate promising antioxidant and anti-inflammatory effects in models of neuroinflammation and oxidative stress, yet translation into predictable CNS uptake remains uncertain [440,441,442]. Thus, PDEVs occupy a unique space: safe, scalable, and bioactive, but require deeper mechanistic insight before they can be positioned as reliable neurotherapeutic vectors [433,439,443].

### 8.3. Synthetic Mimetics

Synthetic exosome-mimetic vesicles are designed to replicate the communication and delivery roles of natural exosomes while sidestepping their limitations of yield and heterogeneity [444,445]. Strategies include polymersomes with controllable membrane chemistry, membrane-coated nanoparticles that borrow cellular surface markers, and hybrid designs that combine synthetic scaffolds with natural membrane fragments [446,447,448]. Such constructs excel in tunability and scalability, making them better suited for standardized manufacturing compared to their mammalian counterparts [444,445,449].

Yet these advantages come with trade-offs [447,448]. Replacing native membranes often diminishes biocompatibility cues that exosomes naturally provide, raising concerns about immune activation and altered clearance [447,448,449]. Nonetheless, synthetic platforms allow for reproducible incorporation of targeting ligands or exosomal motifs, offering a level of precision that natural vesicles rarely achieve [444,445,446]. Early applications highlight their promise in oncology, regenerative medicine, and CNS targeting [447,450,451]. We now connect models to the endpoints that drive go/no-go decisions.

## 9. Translational Models and Decision-Enabling Endpoints

This section is about choosing models that answer the right question, then pairing them with endpoints that actually de risk translation. We first review in vitro BBB systems, from Transwells to iPSC organoids and chips, and spell out what each can and cannot predict. We then move to in vivo models, where species differences and disease-driven permeability shifts can mislead exposure claims. Next, we define decision grade PK metrics, especially fu,brain unbound fraction in brain tissue (f_u,brain_), K_p,brain_ and K_p,uu,b rain_, because total brain levels can lie. Finally, we cover imaging and biomarker readouts that triangulate delivery, target engagement, and safety in the same experiment.

### 9.1. In Vitro Models

In vitro BBB models remain indispensable as early decision tools in neurotherapeutic development [452,453]. Classic Transwell systems with endothelial monolayers offer simplicity and throughput but often fail to reproduce the restrictive tight junctions of the human BBB [454,455]. Adding astrocytes or pericytes in co-culture improves fidelity, as astrocytic signals reinforce junctional protein expression and better align transendothelial electrical resistance (TEER) values with physiological ranges [453,456,457]. These refinements help distinguish passive permeability from transporter-mediated flux, although limitations in dynamic responses persist [454,458].

Human-induced pluripotent stem cell (iPSC)-derived BBB organoids introduce greater biological relevance by capturing species-specific expression of transporters and efflux pumps [453,459]. However, the lack of standardized differentiation protocols results in variable permeability and metabolic profiles across laboratories [453,460]. Microfluidic BBB-on-chip systems address some of these issues by incorporating shear stress, nutrient gradients, and continuous flow, recapitulating the hemodynamic conditions that shape BBB integrity [454,458,461]. These dynamic constructs offer a closer physiological context but are more technically demanding and costly to implement [458,461].

Benchmarking against in vivo data remains essential [454,461]. Metrics such as apparent permeability (P_app) and TEER are routinely compared to animal and human datasets, yet over- or underestimation of drug transport is common [455,462]. The reported dataset illustrates how carefully calibrated microfluidic models can achieve closer alignment with in vivo permeability coefficients [454,461]. Despite progress, no single in vitro system fully resolves the trade-off between scalability and predictive accuracy, underscoring the need for model selection tailored to the specific decision point in development [452,458].

### 9.2. In Vivo Models and Species Differences

Rodent models remain the workhorse of preclinical neurotherapeutics, offering high-throughput screening, ease of genetic manipulation, and well-characterized disease models [463]. Yet their BBB exhibits greater paracellular leakiness than that of primates, which can overestimate drug penetrance [464,465]. This divergence partly explains why promising rodent data often fail to translate into clinical success [463,466]. Moreover, rodents display transporter expression patterns that differ in both abundance and substrate specificity compared to humans, adding further complexity to predictions of central exposure [466,467,468].

Non-human primates provide the closest approximation of human BBB integrity and regional perfusion characteristics [467,469]. Their barrier tightness, transporter repertoire, and cerebrovascular physiology more closely align with human data, making them critical for late-stage validation [465,470]. However, cost, ethical concerns, and limited availability restrict their widespread use [470]. Adding further complication, disease states reshape barrier permeability: ischemic stroke disrupts endothelial junctions, Alzheimer’s disease alters transporter activity, and glioblastoma induces localized leakiness that changes drug distribution [365,471,472].

These species and disease-dependent differences emphasize the translational gap between model systems and patients [463,464]. Cross-species network analyses and computational integration strategies are increasingly used to bridge this gap, but the fundamental challenge remains: no single in vivo model fully captures the nuances of human BBB physiology [455,473]. Strategic selection and careful benchmarking are therefore essential to guide go/no-go decisions in CNS drug development [455,465].

### 9.3. Quantitative Pharmacokinetics (PK) Endpoints

Quantitative pharmacokinetic endpoints are central to linking drug exposure with CNS activity [474]. The f_u,brain_, defines the pharmacologically active pool, while the brain-to-plasma partition coefficient (K_p,brain_) describes overall distribution across compartments [475]. A more precise index is K_p,uu,brain_, the ratio of unbound brain to unbound plasma concentrations, which reflects true equilibrium between compartments and better predicts central efficacy [475,476]. These parameters guide whether a compound achieves sufficient free concentrations at its target site or is limited by efflux transporters and protein binding [477,478].

Measuring these endpoints remains technically demanding [474]. Microdialysis enables direct sampling of interstitial fluid, offering dynamic readouts of unbound concentrations, but it is invasive and limited to specialized settings [479]. Homogenate binding assays, in contrast, are more accessible but prone to overestimation due to disrupted tissue architecture [476,480]. Cerebrospinal fluid is often used as a surrogate for interstitial concentrations, yet differences in turnover and compartmentalization mean CSF rarely mirrors brain extracellular fluid with high fidelity [481,482]. Regulators increasingly emphasize integration of such quantitative PK endpoints with pharmacodynamic measures, particularly through physiologically based pharmacokinetic and PK–PD models, to inform dose selection and reduce translational uncertainty [482,483,484]. By anchoring drug development in f_u,brain_,, K_p,brain_, and K_p,uu,brain_, researchers can more confidently bridge preclinical data with human predictions and make decision-grade assessments of CNS penetration [476,485].

### 9.4. Imaging and Biomarker Readouts

Imaging has become a cornerstone in evaluating how drugs and nanocarriers navigate the BBB [486,487]. Positron emission tomography (PET) tracers provide sensitive, quantitative assessments of permeability, while magnetic resonance imaging (MRI) with contrast agents captures dynamic leakage and regional perfusion in vivo [486,488]. Recent work extends these approaches to track nanocarrier fate over time, revealing how size, charge, and surface chemistry influence deposition within target regions [487,489]. Such dynamic readouts are invaluable not only for confirming delivery but also for ruling out vascular compromise or off-target accumulation that could cloud efficacy signals [490].

In parallel, biomarker development is beginning to complement and extend imaging [491]. Neuroinflammation markers such as GFAP or ICAM-1 flag astrocytic and endothelial responses, while circulating exosomal signatures hint at brain-specific injury or remodeling processes [492,493]. The true translational power lies in linking imaging and biomarkers simultaneously to efficacy—drug exposure within the intended region—and safety, including the detection of edema or inflammatory activation [494,495]. Emerging strategies combine multimodal imaging with panels of fluid biomarkers, offering a near real-time window into drug delivery, target engagement, and tissue response [491,496,497]. This convergence is setting the stage for decision frameworks that go beyond single endpoints and instead integrate orthogonal readouts to guide go/no-go calls with greater confidence [494,498]. What has actually reached patients? We summarize clinical traction and why certain bets are moving first [486] (Table 7).

## 10. Clinical Landscape and Case Snapshots

### 10.1. Neuro-Oncology

Focused ultrasound (FUS) with microbubbles is the most advanced clinical strategy for transiently opening the BBB in neuro-oncology [514,515,516]. By generating localized acoustic cavitation, FUS temporarily loosens tight junctions, permitting chemotherapeutics to achieve higher intratumoral concentrations than with systemic dosing alone [514,515,516,517]. Early-phase trials in glioblastoma and brain metastases report encouraging safety signals, with most adverse events being transient edema or headaches rather than irreversible damage [515,517]. Imaging-confirmed increases in drug penetration, paired with pharmacokinetic analyses, have strengthened confidence that this approach is technically feasible and biologically impactful [515,517].

Parallel efforts explore ligand-targeted nanocarriers for glioblastoma, including transferrin- and integrin-directed liposomes, which are designed to selectively home to tumor vasculature or infiltrating glioma cells [518,519,520]. These systems aim not only to improve local accumulation but also to minimize systemic exposure [518,519]. Key endpoints now extend beyond radiographic progression-free survival to include intratumoral drug levels, pharmacodynamic signatures, and radiomic biomarkers that track response heterogeneity [289,515,516]. Yet regulatory progress remains uneven. Enrollment in neuro-oncology trials frequently lags behind projections, with disparities in infrastructure and patient access limiting study completion rates [521,522]. As adaptive designs and external control datasets gain traction, the field is moving toward more flexible, inclusive trial frameworks capable of sustaining momentum in a disease space with urgent unmet needs [516,521,522].

### 10.2. Neurodegeneration

Alzheimer’s disease trials have tested diverse delivery routes, with intranasal insulin standing out for its ability to bypass systemic metabolism and provide direct brain access [523,524]. Peptide- and polyphenol-loaded carriers are also under investigation to stabilize bioactive molecules while enhancing their penetration into hippocampal and cortical regions [525,526]. Although some studies report cognitive benefits and favorable biomarker shifts, variability in patient populations and endpoint sensitivity continues to limit clear conclusions [527,528,529]. Reliance on radiographic and cognitive scales alone often underestimates subtle, early effects, underscoring the need for multimodal biomarker panels [527,528,530].

In Parkinson’s disease, dopamine prodrugs and nanoparticle-based formulations represent strategies to extend half-life and reduce peripheral toxicity while restoring striatal dopamine tone [163,531,532]. Several trials show encouraging motor improvements, yet variability in absorption and BBB transport remains a barrier to consistency [531,532,533]. Lessons from both Alzheimer’s and Parkinson’s pipelines converge on the importance of robust biomarkers, sensitive endpoints, and trial designs that accommodate disease heterogeneity [527,528,530]. Without these refinements, even promising therapeutic concepts risk falling short in translation [527,530,534].

### 10.3. Psychiatric and Pain Indications

Early translational studies in psychiatry and pain have focused on phytochemicals such as curcumin and resveratrol, as well as terpenoids such as pinene and linalool, which show preclinical promise for mood regulation and analgesia [35,535,536]. Yet progress into robust clinical validation remains limited [35,535]. Subjective endpoints and the strong influence of placebo responses complicate signal detection, while modest funding and heterogeneous trial designs further slow momentum [537,538]. Current exploratory efforts in depression and chronic pain increasingly employ advanced delivery systems and biomarker-informed approaches, but most remain proof-of-concept [539,540,541,542]. The field illustrates both opportunity and fragility in translating natural compounds into psychiatric and pain therapeutics [35,535,543,544].

### 10.4. Snapshot

First-in-human studies with natural compounds illustrate both opportunity and limitation across therapeutic domains. Oncology has the deepest record, with plant-derived chemotherapeutics and semi-synthetic derivatives advancing into late-stage trials [545,546]. Yet many candidates stall due to safety uncertainties, inconsistent batch quality, or regulatory concerns related to chemistry and manufacturing controls [547]. In neurodegeneration, compounds such as curcumin, resveratrol, and quercetin have entered clinical testing, often showing bioactivity but hampered by poor bioavailability and heterogeneous outcomes [24,548,549]. Psychiatric indications remain the least mature, with strong preclinical rationale but scarce head-to-head trials against approved therapies [550,551].

This comparative landscape highlights clear gaps for phytochemicals: translation remains fragmented, efficacy signals are often modest, and reproducibility suffers without rigorous manufacturing standards [546,547]. The field now recognizes that scientific novelty alone is insufficient. Translation hinges on safety, manufacturability, and regulatory clarity—complex systems demand disciplined CMC [551] (Table 8).

## 11. Material Safety and Immunogenicity

### 11.1. Hemolysis, Complement Activation, Microglial Responses

Early material safety screening hinges on blood compatibility, because initial interactions with blood components often dictate downstream immune trajectories [566,567]. Hemolysis is not a benign artifact but an active trigger of innate immunity [568,569]. Cell-free heme and heme-bearing microvesicles directly activate the complement cascade, driving C3 cleavage, leukocyte activation, and cytokine release, thereby linking red blood cell damage to acute inflammatory toxicity and organ injury [568,569,570]. As a result, hemolysis assays are most informative when paired with measurements of complement split products and early cytokines in serum or whole-blood systems [566,570,571]. Across preclinical and clinical contexts, rising immune complexes, C3a generation, or depletion of C3 and C4 consistently correlate with infusion reactions and dose-limiting hypersensitivity, particularly during dose escalation [568,571,572].

For CNS-targeted materials, microglia and astrocytes represent a distinct and highly sensitive safety axis [117,573,574]. Complement opsonization can promote microglial uptake that is either neuroprotective or deleterious, depending on persistence and inflammatory tone [573,574]. Astrocyte-derived complement components, together with IL-1, TNF, and IL-6 signaling, shape microglial activation states and synaptic integrity [574,575,576]. Here, physicochemical parameters act as immune dials rather than binary switches [566]. Smaller size, higher dose, and increased positive surface charge enhance uptake and cytokine release, while excessive activation pushes glia toward chronic inflammatory phenotypes [576,577,578]. Early integration of these variables helps distinguish immunologically silent designs from those primed to provoke neuroimmune risk [577,578,579].

### 11.2. Hemocompatibility and Neuroinflammation Assays

Standardized hemocompatibility testing remains the first safety filter for blood-contacting and intravascular materials [580,581,582]. In vitro panels aligned with ISO 10993-4 [583] typically assess platelet adhesion and aggregation, the intrinsic and extrinsic coagulation pathways, and complement activation, using thrombin generation, aPTT, platelet surface markers, and C3a or C5b-9 formation. [580,582,584]. Sequential whole-blood and platelet-rich plasma assays increasingly capture the cascade from protein adsorption to thrombogenicity and cytokine release, allowing mechanistic interpretation rather than binary pass-fail outcomes [580,585].

Neuroinflammation assays extend this logic into the CNS space. Human iPSC-derived microglia and astrocytes, cocultures, and emerging organoid systems enable multiplex cytokine profiling, complement C3 readouts, and neurotoxicity markers under controlled stimuli [586,587,588]. Critically, aligning these outputs with clinically validated biomarkers such as GFAP, IL-6, or TNF strengthens the bridge between in vitro signals and patient-level neuroinflammatory risk [589,590,591,592].

### 11.3. Biodistribution and Clearance

Biodistribution and clearance represent a central determinant of both efficacy and long-term safety for material-based therapeutics [593,594]. Following systemic administration, the majority of nanoscale materials are rapidly sequestered by the mononuclear phagocyte system, with liver and spleen often capturing most of the injected dose [595]. Uptake by Kupffer cells, splenic macrophages, and sinusoidal endothelium can markedly reduce target tissue exposure while establishing persistent intracellular reservoirs [595,596]. Such retention may remain clinically silent, yet it raises concerns under repeated dosing and complicates the interpretation of chronic toxicity risk [595,597].

Design choices strongly bias this balance between persistence and elimination [593,598]. Ultrasmall or biodegradable architectures favor renal or hepatobiliary clearance, shortening organ residence while preserving therapeutic exposure [594,599]. In contrast, larger or rigid constructs tend toward lysosomal trapping [593,600]. De-risking strategies increasingly combine biodegradable scaffolds, surface chemistry optimization, and dose fractionation to limit cumulative burden without sacrificing pharmacological performance [601,602,603].

### 11.4. Chemistry, Manufacturing, and Controls (CMC)/Carrier-Mediated Transport (CMT) and Critical Quality Attributes (CQAs)

Critical quality attributes anchor the translation of complex materials from bench to clinic [604,605]. Across quality by design frameworks, particle size, polydispersity, zeta potential, encapsulation efficiency, and release kinetics consistently emerge as core CQAs because they integrate manufacturability with exposure and immunogenicity risk [604,606]. Multivariate and machine learning driven designs show that modest shifts in process parameters can propagate into meaningful changes in these attributes, with downstream effects on stability and biological performance [607,608,609]. Release profiles, often first-order or diffusion-controlled, are increasingly treated as quantitative CQAs rather than descriptive outcomes [604,610].

For CNS administered products, sterility and endotoxin control are nonnegotiable [611,612]. Endotoxin thresholds are substantially lower than for systemic routes, reflecting heightened neuroinflammatory sensitivity [613,614]. Routine lot release; therefore, couple-validated BET or rFC assays with conservative specifications aligned with intrathecal exposure [613,615]. Stability programs add another layer of complexity [616,617]. Aggregation, content leakage, and loss of redispersibility during storage or lyophilization can silently erode CQAs unless cryoprotectants and freezing protocols are optimized [617,618].

GMP alignment ultimately depends on reproducibility [619,620]. To strengthen translational relevance, we note that the most frequently reported platforms, PLGA-based nanoparticles, liposomes, SLNs or NLCs, and nanoemulsions differ sharply in scale up risk [400]. Emulsion and precipitation routes can be sensitive to mixing energy, solvent removal, and raw material variability, so scale up should be discussed in terms of process controls rather than nominal composition [621]. In contrast, clinically mature unit operations, including high pressure homogenization for lipid systems and validated solvent evaporation or microfluidic mixing for polymeric systems, tend to offer clearer control strategies [622]. We therefore emphasize that near term candidates are those that preserve size and PDI, loading, and release kinetics under scale up, with stability, sterility, and endotoxin specifications maintained at lot release. Batch to batch fidelity in physicochemical attributes, sterility, and potency transforms CMC data from descriptive characterization into a predictive safety framework [620,623]

### 11.5. Regulatory Expectations

Regulatory agencies approach nanomedicines through a risk-based, case-by-case lens that reflects their structural diversity and evolving biology [624,625]. Both the FDA and the EMA emphasize nanomedicine-specific risks that extend beyond those of conventional small molecules, including altered biodistribution, immune activation, and long-term tissue persistence [624,626]. Guidance increasingly calls for deeper physicochemical characterization, nano-relevant immunotoxicity testing, and justification when standard ICH assays lack sensitivity [627,628]. For complex biological nanoparticle hybrids such as lipid nanoparticles, polymer conjugates, or gene delivery systems, regulators treat products as non-biological complex drugs, limiting assumptions of equivalence and requiring product-specific clinical evidence [624].

Bridging preclinical data to first-in-human studies relies on standardized safety frameworks that integrate in vitro and ex vivo human blood assays with human blood, and targeted in vivo assays [627,629]. For CNS indications, expectations tighten further [630]. BBB interactions, neuroinflammation risk, and irreversible outcomes demand a transparent risk–benefit narrative grounded in mechanistic data rather than exposure alone [628,630]. Data science tightens design loops and right-sizes risk before first dose in humans [631,632].

## 12. Data Science, Modeling, Artificial Intelligence (AI)-Guided Design

### 12.1. BBB Permeability Prediction and Polypharmacology

Data science increasingly reframes CNS design from intuition to prediction [633,634] QSAR and machine learning models trained on large BBB datasets now capture both quantitative logBB and categorical permeability with accuracy that supports early triage [633,634]. Beyond simple lipophilicity, modern models incorporate nonlinear descriptors and explicitly account for transporter effects, with P-gp emerging as a dominant determinant of CNS variability [153,635]. This is especially relevant for polyphenols, where favorable passive diffusion can be offset by strong efflux liability [153]. Transporter-aware modeling, combined with PBPK frameworks, allows permeability to be interpreted as a balance of influx and clearance rather than a static property [635,636].

AI-guided polypharmacology further expands this view [637,638]. Network-level profiling distinguishes harmful off target promiscuity from coordinated multi target engagement, enabling rational exploitation of pleiotropic mechanisms that are often intrinsic to natural products and CNS therapeutics [639,640].

### 12.2. Multi-Objective Formulation Optimization

Multi objective optimization reframes formulation design as a data driven negotiation between competing constraints [641]. Machine learning models trained on design of experiment data now predict how size, zeta potential, and drug loading jointly shape potency, exposure, stability, and manufacturability [642,643]. Rather than chasing a single optimum, Bayesian and evolutionary algorithms explore Pareto fronts, revealing trade-offs that are invisible to one-factor-at-a-time approaches [641,644]. In practice, this enables probabilistic design spaces where acceptable formulations are defined by balanced desirability rather than maximal performance [642]. Such frameworks accelerate iteration, reduce experimental burden, and align early formulation choices with downstream safety and GMP feasibility [642,643].

### 12.3. Physiologically Based Pharmacokinetic (PBPK)/Pharmacokinetic–Pharmacodynamic (PKPD) and Digital Twins

Physiologically based pharmacokinetic modeling has become a cornerstone for forecasting CNS exposure and BBB penetration in silico [636,645]. Modern CNS PBPK platforms resolve regional brain compartments, passive permeability, and active efflux, allowing human predictions to be extrapolated from limited preclinical or in vitro data [645,646]. Coupling these frameworks to PK PD models refines dose response by linking brain time courses to target engagement and effect kinetics, enabling virtual dose fractionation before first exposure [635]. Digital twin concepts extend this logic further [647]. By integrating PBPK, machine learning derived BBB parameters, and virtual populations, individualized predictions of permeability and response become feasible [645,647]. We synthesize the major gaps and convert them into concrete, testable strategies.

## 13. Research Gaps and Concrete Strategies

### 13.1. Standardized Human-Relevant Pharmacokinetics (PK) Endpoints

A clear gap is the lack of standardized, human-relevant CNS PK endpoints that translate cleanly from animals to early clinical trials. Across conceptual surveys and candidate-selection frameworks, K_p,uu,brain_ repeatedly emerges as the most defensible common currency because it captures BBB transport and binding within an unbound metric. Yet human K_p,uu,brain_ data remain sparse, and many programs still rely on total brain concentrations or non-comparable surrogates. A concrete strategy is universal adoption of K_p,uu,brain_, paired with PBPK-informed target-site exposure ratios that connect unbound concentrations to in vivo IC50-class benchmarks and pharmacodynamic effect.

Methodologically, the field needs harmonization of how K_p,uu,brain_ and related endpoints are measured in humans. Combined PET plus microdialysis can convert imaging signals into unbound interstitial exposure, while mechanistic PBPK platforms can reconcile compartmental and spatial heterogeneity when CSF is unreliable. Standardized PET endpoints for exposure and engagement, together with aligned CSF metrics such as AUC, Cmax, and Ctrough normalized to potency, would make datasets interoperable. Regulatory pressure could then drive CNS drug development toward quantifiable, auditable endpoints, replacing subjective “brain penetration” claims with decision-grade measures.

### 13.2. Humanized Blood–Brain Barrier (BBB) Models with Disease Fidelity

Humanized BBB models still fall short when they trade biological realism for convenience. A priority gap is scaling iPSC-derived BBB organoids, spheroids, and self-assembled microvessels into flow-conditioned systems that reproduce shear, polarization, and transport kinetics seen in vivo. Microfluidic BBB-on-a-chip platforms and perfusable 3D microvessels now achieve low paracellular permeability and strong junctional phenotypes, yet protocol and donor variability remain major sources of noise [648].

Concrete strategy: Build disease fidelity into the neurovascular unit, not just the endothelium. Co-culture designs that incorporate pericytes, astrocytes, neurons, and microglia, ideally from patient or isogenic iPSC backgrounds, capture inter-individual differences in maturation, transporter function, immune cell trafficking, and barrier breakdown [649]. The field then needs rigorous validation against human in vivo benchmarks, such as PET permeability proxies, CSF-to-plasma relationships, and clinical drug exposure patterns, so these models stop being “pretty biology” and start reducing translational attrition.

### 13.3. Prodrug Translation Playbook

A translational prodrug playbook has to start with transporter rigor, not transporter “positive” checkboxes. For carrier-mediated designs, uptake should be quantified with K_m_ and V_MAX_ under physiologic substrate conditions, then stress tested for competition with endogenous ligands and likely co medications [347]. Pharmacoproteomic transporter expression can anchor these kinetics to realistic barrier capacity, while time course uptake modeling helps separate true transported substrates from high affinity binders that never meaningfully cross.

Next, de-risk activation and safety in parallel. Cleavage mapping should quantify where and how fast the promoiety is removed across plasma, liver, brain microvessels, parenchyma, and disease relevant compartments to enforce brain first activation and avoid premature systemic unmasking. Off target profiling can be expanded beyond cell lines using tissue thermal proteome profiling or ABPP style probes across organ panels [650]. Go/no go rules then become tangible: require a brain-unbound exposure gain, a defined brain to plasma activation ratio, no dominant peripheral off target signals, and a pharmacodynamic effect that tracks brain exposure in 3D GBM models or organotypic brain slices.

### 13.4. Long-Term Safety and Immunogenicity Registries

Long-term safety remains a blind spot when CNS trials end at symptom curves rather than at biology and latency. A concrete strategy is post-trial registries that follow participants for years, capturing delayed toxicities, immune responses, and neuroinflammation through linked EHR and claims data, structured adverse event reports, and longitudinal fluid or imaging biomarkers such as GFAP, YKL-40, sTREM2, or neuroinflammatory PET [651]. These registries should interlock with pharmacovigilance databases via standardized, FAIR data models and privacy preserving linkage, so signals can be detected, replicated, and risk managed across systems. Biologics and cell or gene therapies offer the template: mandated long follow up, harmonized reporting, and global registries that turn rare late events into quantifiable risk [651].

### 13.5. Manufacturability and Quality Control (QC) for Complex Carriers

A central translational gap for complex nanocarriers is that manufacturability and quality control (QC) often lag behind formulation ingenuity. quality by design (QbD) should be treated as the organizing logic, starting with a clear quality target product profile (QTPP) and mapping CQAs to CMAs and CPPs so that a justified design space and control strategy survive scale-up [604]. Yet non-linear formulation process couplings and raw material drift still drive lot-to-lot variability, especially for surface functionalized systems where small chemistry changes reshape size, charge, corona, and bioactivity. Concrete fixes include PAT-enabled real-time monitoring (inline or online size sensing, turbidity, spectroscopy, multivariate analytics), semi-continuous or continuous lines, and tighter incoming material specifications plus stage-gated in-process controls to secure reproducible release quality [652].

### 13.6. Clinical Trial Design Upgrades

Clinical trials for BBB therapeutics need design upgrades that treat BBB heterogeneity as a core covariate rather than background noise. Adaptive platform, basket, and window-of-opportunity approaches can rapidly prune futile delivery strategies while learning which BBB modulation, timing, and dosing actually shift brain exposure [653]. Pair this with enrichment: stratify participants by BBB integrity or permeability status using DCE MRI, PET-based uptake metrics, or fluid markers reflecting barrier leakage and clearance kinetics.

Endpoints should prove target engagement, not just clinical change [654]. Imaging derived cerebral PK, longitudinal PD imaging, and permeability limited PBPK models can define exposure response relationships and justify go or no-go decisions. To satisfy regulators for high-cost, high-complexity products, adaptations and estimands must be pre specified, bias controlled, and CMC and companion diagnostics aligned early. Finally, we outline what will likely materialize soon and what needs deeper tech maturation,

## 14. Roadmap: Short-Term vs. Long-Term

### 14.1. Short-Term (2–4 Years)

In the short term (2 to 4 years), the most “deployable” polyphenol programs will likely be ligand-targeted PLGA nanoparticles and liposomes carrying resveratrol and curcumin, chosen because their safety narratives are mature while formulation science can add real value. Curcumin has a clearer clinical signal for nanocarrier translation, including an ongoing early phase study of intravenous liposomal curcumin in high grade gliomas. By contrast, most resveratrol nanoformulations cited in this review remain preclinical, so we now frame resveratrol as a near term candidate for formulation refinement rather than as a platform already validated in Phase II CNS trials [655]. Practical targets include transferrin or RVG-style ligands for BBB facing delivery, plus dual loading to exploit complementary redox and anti-inflammatory pharmacology. The translation gate is not efficacy hype, it is reproducible particle size, drug loading, and stability under scalable unit operations [171].

Intranasal mucoadhesive nanoemulsions and nanoemulgels are even closer to early-phase readiness because dosing ergonomics can be engineered into a sprayable, residence-time-extending product. Chitosan-coated or thermotriggered in situ gel formats already map nicely onto trial-friendly endpoints: nasal tolerability, systemic exposure, and nose-to-brain PK surrogates such as regional brain concentrations in imaging-rich substudies or CSF exposure when justified [190]. Here, the CMC control strategy must be well defined, focusing on droplet size distribution, rheology, spray plume metrics, and preservative compatibility.

LAT1-anchored prodrugs are the “biology first” option. The appeal is a validated transporter with design rules for aromatic promoieties and linker choices, enabling higher brain exposure with lower peripheral burden [346,402]. Short-term success will come from leveraging known promoiety scaffolds, building a screening cascade that confirms LAT1 affinity, bioconversion kinetics, and intra brain distribution, then anchoring dose selection to target engagement readouts in neurons and glia.

**Figure 4:**
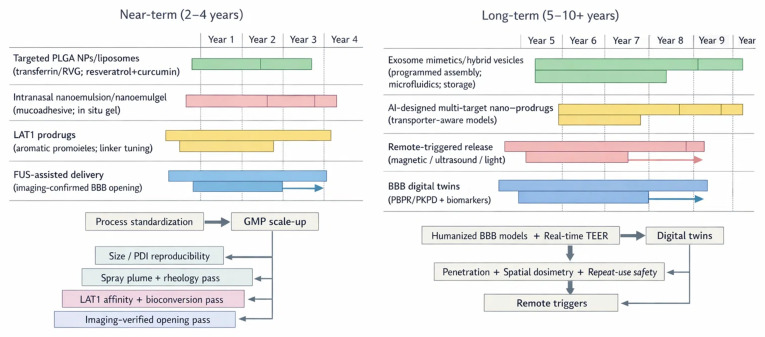
Roadmap timeline for brain delivery platforms: short-term deployables versus long-term maturation, with validation gates and dependencies. Gantt-style bands summarize expected maturation windows for CNS delivery strategies and the dependencies that determine whether programs advance from feasibility to translation. Short-term (2–4 years) tracks emphasize “deployable” options with the clearest CMC and endpoint pathways: ligand-targeted PLGA nanoparticles/liposomes (e.g., transferrin or RVG; resveratrol/curcumin, including dual loading) gated by reproducible size/PDI, loading, and stability under scalable unit operations; intranasal mucoadhesive nanoemulsions/nanoemulgels (chitosan or thermo/in situ gels) gated by droplet-size distribution, rheology, spray plume metrics, preservative compatibility, and tolerability plus PK surrogates (CSF or imaging-rich substudies where justified); LAT1-anchored prodrugs gated by LAT1 affinity, bioconversion kinetics, intrabrain distribution, and target engagement in neurons/glia; and focused ultrasound-assisted regional delivery gated by imaging-confirmed BBB opening, local PK confirmation, and regulator-recognized safety monitoring. Longer-term (5–10+ years) bands capture exosome mimetics/hybrid vesicles (GMP scale-up and heterogeneity control), AI-designed multi-target nano–prodrugs (auditable datasets and prospective validation), remote-triggered release (dosimetry and repeat-use safety standards), and BBB “digital twins” integrating PBPK/PKPD with real-time TEER and registries. AI, artificial intelligence; BBB, blood–brain barrier; CMC, chemistry, manufacturing, and controls; CNS, central nervous system; CSF, cerebrospinal fluid; GMP, good manufacturing practice; LAT1, large neutral amino acid transporter 1; PBPK, physiologically based pharmacokinetic; PDI, polydispersity index; PK, pharmacokinetics; PKPD, pharmacokinetic–pharmacodynamic; PLGA, poly(lactic-co-glycolic acid); RVG, rabies virus glycoprotein; TEER, transendothelial electrical resistance.

Focused ultrasound-assisted regional delivery fits the same horizon when paired with drugs that already have a clinical path, such as chemotherapy for glioma margins or neuroprotectives with clean systemic safety profiles. It offers a controllable exposure window, but only if trials pre-specify imaging-based BBB opening, local PK confirmation, and safety monitoring that regulators recognize [656]. Across all these tracks, feasibility wins: scalable manufacturing, release tests that predict performance, and endpoints that prove delivery plus mechanism, not just symptomatic change [657] (Figure 4).

### 14.2. Longer-Term (5–10+ Years)

Looking 5 to 10 years out, exosome mimetics and hybrid vesicles could become the “biomimetic workhorses” of brain delivery, but only if GMP-scale-up stops being artisanal. The roadmap points to programmed assembly, extrusion, and liposome fusion approaches, followed by process intensification via microfluidics and bottom-up manufacturing to reduce heterogeneity while improving yield [658]. Cost control will hinge on standardized membrane sourcing, robust cargo-loading metrics, and shelf-stable storage protocols.

In parallel, AI-designed multi target nano prodrugs may unlock rational polypharmacology, not by adding more ligands, but by learning which combinations actually cooperate at the BBB. The key upgrade is transporter awareness in silico: prediction stacks that integrate passive permeability, efflux risk, and carrier or promoiety interactions with uptake transporters [659]. Regulatory credibility will depend on curated datasets, auditable models, and prospective validation rather than retrospective fits.

Remote-triggered release platforms promise precision with fewer systemic side effects, such as magnetic fields [660].

Finally, patient specific BBB digital twins could connect PBPK and PKPD to biomarker-informed adaptation, turning trial dosing into a learning loop [659]. That vision depends on humanized BBB models with functional readouts, such as real-time TEER, longitudinal safety registries for complex nanomedicines, and early alignment with regulators on what counts as validated exposure and engagement evidence [661] (Figure 4).

## 15. Clinical Applications and Translational Implications

The concepts synthesized in this study have direct clinical relevance for the development of next-generation neurotherapeutics targeting disorders with high unmet medical need, including neurodegenerative diseases, neuropsychiatric conditions, epilepsy, and brain tumors [662]. By systematically linking the physicochemical limitations of plant-derived compounds to rational delivery solutions—such as nanocarriers, transporter-targeted prodrugs, intranasal administration, and device-enabled BBB modulation—this framework provides actionable guidance for improving CNS drug exposure where conventional pharmacotherapy has failed [4]. In clinical contexts characterized by multifactorial pathophysiology, such as Alzheimer’s disease, Parkinson’s disease, depression, and chronic pain, phytochemicals with pleiotropic anti-inflammatory, antioxidant, and neuromodulatory actions may offer therapeutic advantages if reliable brain delivery can be achieved [663].

From a translational standpoint, the study supports a shift away from empiric compound selection toward delivery-first clinical development, in which candidate molecules are paired early with route, carrier, or prodrug strategies to achieve decision-grade CNS exposure [72]. Clinically, this approach may enable dose reduction, improved safety margins, and more predictable pharmacokinetics, particularly in vulnerable populations such as older adults or patients receiving polypharmacy [664]. Furthermore, the discussed platforms—especially intranasal delivery and focused ultrasound–mediated BBB opening—offer opportunities for region-specific or noninvasive treatment paradigms, which are increasingly relevant in precision neurology and psychiatry [665]. Collectively, these insights inform the design of early-phase clinical trials, guide biomarker and endpoint selection, and support regulatory-aligned translation of plant-derived neurotherapeutics from bench to bedside [666].

Early clinical traction is currently strongest for approaches that are already compatible with hospital workflows [667]. Focused ultrasound plus microbubbles has entered human studies across glioma and neurodegeneration, with imaging-confirmed BBB opening and repeat-session feasibility forming the core evidence base [668]. Intranasal delivery also has clinical precedents in neurology and psychiatry, although formulation sensitivity and dosing variability remain recurrent limitations [669]. In contrast, ligand-decorated nanocarriers and multifunctional hybrid systems are still dominated by preclinical datasets, so we now frame them as development-stage technologies rather than as broadly validated clinical solutions [669]. A consolidated view of these clinical signals is summarized in Table 8.

## 16. Conclusions and Translational Implications

RMT targeting via intranasal delivery enables potent, stable payloads to exploit the nose-to-brain pathways [161]. Focused ultrasound with microbubbles provides a reversible, local window of BBB opening [670]. Exosome mimetics and nano prodrugs stay promising but remain unproven. We posit that translation will accelerate when workflows centered on K_p,uu,brain_ are adopted as a shared quantitative standard across disciplines. This framework treats delivery as a coupled system in which chemistry, carrier design, route selection, and exposure measurement are co-optimized. Start with a liability map for each phytochemical, then choose the simplest strategy that can raise unbound brain exposure while meeting safety and chemistry, manufacturing, and controls constraints. Report f_u,brain_, unbound plasma fractions (f_u,p_), and K_p,uu,brain_, then connect them to target engagement, imaging, and functional outcomes. Future research should deliver head-to-head platform comparisons on identical payloads, validate human-relevant BBB models against in vivo benchmarks, and build PBPK guided dose projections that survive species shifts. Methodologically, the field also needs harmonized critical quality attributes and longitudinal safety panels that capture complement activation, microglial priming, and vascular repair. Done right, these principles can accelerate barrier-limited therapeutics far beyond phytochemicals.

## Figures and Tables

**Table 1:** BBB transport machineries and implications for phytochemicals. This table summarizes the dominant BBB transport routes relevant to plant-derived neurotherapeutics and links each route to pragmatic design levers. Pathways are framed as decision levers: passive diffusion is constrained by physicochemical liabilities common in polyphenols; carrier-mediated transport and receptor-mediated transcytosis provide engineered influx opportunities; adsorptive and cell-penetrating peptide strategies can amplify uptake but trade specificity for risk; and efflux pumps (P-gp, BCRP, MRPs) often set the ceiling for unbound brain exposure even when in vitro permeability appears promising.

Pathway	Molecular Prerequisites	Exemplars	Impact on Phytochemicals	Engineering Lever(s)	References
Paracellular diffusion (tight junction-limited)	Effectively negligible at an intact BBB; requires transient junction loosening or pathological leak	Small hydrophiles in disease-associated leak states	Native polyphenols remain largely excluded; leak is disease- and region-dependent and poorly controllable	Localized opening approaches (e.g., focused ultrasound with microbubbles); avoid programs that depend on nonspecific leak	[67,68,69]
Transcellular passive diffusion	Small size, low polarity, limited H-bonding; favorable lipophilicity; minimal efflux liability	CNS-permeable small molecules; selected alkaloids	Many phytochemicals exceed polarity and H-bonding windows; metabolism and efflux can negate apparent permeability	Prodrug or soft-drug design; tune logD and polar surface area; stabilize against first-pass metabolism; solubility-enabling formulations	[70,71,72]
Carrier-mediated transport (CMT)	Structural mimicry of endogenous nutrients; transporter affinity plus adequate chemical stability	GLUT1 (glucose), LAT1 (large neutral amino acids), MCTs (monocarboxylates)	Provides an influx handle for polar phytochemicals, but competition with endogenous substrates and species differences can limit delivery	Transporter-hijacking prodrugs (amino acid, glucose, monocarboxylate promoieties); K_m_/V_MAX_-aware design; brain-selective cleavage	[73,74,75]
Receptor-mediated transcytosis (RMT)	Ligand engagement within a productive affinity window; excessive avidity increases sequestration and lysosomal routing	Transferrin receptor, insulin receptor, LRP1 (targeting designs)	Enables macromolecular and nanoparticle shuttling, but ligand density and valency control release into brain parenchyma	Ligand-decorated nanocarriers; optimize affinity and ligand density; cleavable linkers; designs that favor recycling over degradation	[58,76,77]
Adsorptive-mediated transcytosis and CPP uptake	Net positive charge and/or CPP motifs; electrostatic interactions with endothelial glycocalyx	Tat, penetratin, RVG-derived peptides (as CPP/targeting motifs)	High uptake can trade specificity for off-target accumulation and cytotoxicity; “more cationic” is not always better	Charge-switchable coatings; stimulus-unmasking CPPs; cap surface charge; combine with targeting ligands to improve selectivity	[58,72,76]
Active efflux (ABC transporters)	Substrate recognition by ATP-driven pumps; efflux can dominate even when passive permeability is favorable	P-gp, BCRP, MRPs	A key barrier for many polyphenols; inhibition or induction can shift CNS exposure unpredictably across age, disease, and comedication	Efflux-evading prodrugs; corona control and stealth coatings; carrier strategies that reduce free substrate at the luminal membrane; early efflux liability screening	[70,71,72]

ABC, ATP-binding cassette; BBB, blood–brain barrier; BCRP, breast cancer resistance protein; CMT, carrier-mediated transport; CNS, central nervous system; CPP, cell-penetrating peptide; GLUT1, glucose transporter 1; K_m_, Michaelis constant; LAT1, L-type amino acid transporter 1; LRP1, low-density lipoprotein receptor-related protein 1; MCTs, monocarboxylate transporters; MRPs, multidrug resistance-associated proteins; P-gp, P-glycoprotein; RVG, rabies virus glycoprotein; RMT, receptor-mediated transcytosis; V_MAX_, maximum transport rate.

**Table 2:** Translational map from phytochemical class to key delivery liabilities and practical enabling strategies for CNS development across BBB-constrained programs. Phytochemical scaffolds share recurring developability bottlenecks at the BBB, yet the dominant liability differs by class. The table links representative compounds discussed in the manuscript to the most common physicochemical and biopharmaceutical constraints, then pairs each class with a preferred enabling strategy that is compatible with scale-up and safety screening.

Class	Examples	Main Delivery Hurdles	Most Useful Enabling Strategy	Practical Notes	References
Polyphenols	Resveratrol, quercetin, curcumin	Phase II metabolism, efflux, low solubility, chemical instability	Prodrug or transporter-targeted promoieties; nanoencapsulation (polymeric NPs, liposomes, SLNs); consider intranasal only when justified	Often light and pH sensitive; phenolic acids can ionize depending on context; bitter or astringent taste may limit adherence	[155,156,157]
Alkaloids	Berberine, galantamine	Ionization plus transporter effects, variable oral bioavailability, efflux variability, CYP interactions	Salt selection plus lipid carriers or micelles; prodrug or carrier shielding; controlled release or alternate routes	Typically basic pK_a_ so cationic at physiological pH; strong bitterness; potency can partly offset limited brain partitioning	[116,122,155]
Terpenoids	Ginkgolides, pinene, linalool	Very low aqueous solubility, volatility, oxidative degradation, high binding plus rapid metabolism	Self-emulsifying systems, nanoemulsions, cyclodextrins, lipid nanoparticles; intranasal for rapid onset when appropriate	Mostly neutral; strong aroma and taste; check irritation risk for concentrated essential-oil-type components	[155,157,158]
Cannabinoids	CBD, THC	High interindividual variability, extensive metabolism, drug interactions, long tissue residence; THC psychoactivity and regulation	Lipid vehicles or nanoemulsions; polymeric carriers or depots; route optimization and dose fractionation to reduce peak effects	Light and oxygen sensitive; very lipophilic and largely neutral; legal and labeling constraints can shape trial design	[159,160,161]

BBB, blood–brain barrier; CBD, cannabidiol; CNS, central nervous system; CYP, cytochrome P450; NPs, nanoparticles; pK_a_, acid dissociation constant; SLNs, solid lipid nanoparticles; THC, Δ9-tetrahydrocannabinol; UGT, UDP-glucuronosyltransferases.

**Table 3:** Nanocarrier platforms for BBB-constrained phytochemicals: design levers, translational performance, and CMC/GMP-critical considerations. This table offers a decision-oriented snapshot of major nanocarrier platforms for brain delivery, linking each to core/shell materials, surface strategies (PEG, receptor ligands, corona control), loading modes, and release logic. Release is categorized as constitutive (diffusion/erosion) or stimulus-enabled (pH/redox/enzymes; external triggers such as magnetic fields or focused ultrasound). Key trade-offs include leakage versus retention (liposomes), payload expulsion (SLNs), reproducibility/stability limits (nanoemulsions), multivalency versus toxicity (dendrimers/CPP-like surfaces), long-term safety uncertainty (inorganic systems), and batch heterogeneity/regulatory issues (exosomes). CMC/GMP notes emphasize quality by QbD priorities: size/PDI, zeta potential, encapsulation efficiency, release kinetics, sterility/endotoxin, and scalable manufacture.

Platform	Best for	Key Constraint	Typical Release or Trigger	CMC Priority	Example Payloads	Refs
Polymeric NPs (PLGA, PEG-PLGA; chitosan hybrids)	Hydrophobic small molecules; sustained delivery	MPS uptake; burst release if not tuned	Diffusion plus polymer erosion; optional pH or redox modules	Size and PDI, zeta potential, loading, release kinetics, sterility and endotoxin, scale-up reproducibility	Resveratrol, curcumin, flavonoids; intranasal chitosan–PLGA example: gemcitabine	[66,235,241]
Liposomes (PEGylated, ligand-decorated)	Mixed hydrophilic and hydrophobic cargo; modular targeting	Leakage and shelf-life limitations	Baseline leakage; optional thermo- or pH-responsive designs	Lipid composition controls, leakage stability, filtration-compatible sterility, lyophilization strategy	Resveratrol, curcumin; peptide cargos	[236,237,242]
SLNs and NLCs	Lipophilic phytochemicals; controlled release	SLN loading limits; storage-driven expulsion reduced in NLCs	Diffusion from lipid matrix; matrix reorganization effects	Lipid polymorphism, surfactant system, storage stability, size and PDI, loading and expulsion monitoring	Polyphenols, terpenoids; chronic neurodegeneration formulations	[243,244,245]
Nanoemulsions and nanoemulgels (often intranasal)	Fast nose-to-brain uptake; solubilization of hydrophobes	Reproducibility, aggregation, shelf-life challenges	Rapid absorption; gels extend residence time via thermo or ion response	Droplet-size distribution, rheology for gels, spray plume and metered dosing, preservative compatibility, long-term stability	Curcumin, resveratrol, terpenoids; cannabinoid-class lipid vehicles	[242,246,247]
Dendrimers (PAMAM, carbosilane)	Programmable multivalency; peptides and nucleic acids	Cationic toxicity and clearance trade-offs	Cleavable linkers for conjugates; diffusion for encapsulated cargo	Generation control, residual monomers and solvents, substitution ratio, sterility and endotoxin, charge-linked immunotoxicity screening	Peptides; nucleic acids such as siRNA-class cargos	[238,239,240]
Polymeric micelles	Solubilization of poorly soluble compounds; triggerable release	Dilution-driven disassembly in vivo	pH or enzyme destabilization triggers	Size and PDI, critical micelle concentration, loading and release, serum stability, storage and lyophilization effects	Hydrophobic phytochemicals; siRNA delivery examples in glioblastoma context	[235,239,240]
Inorganic or carbon nanostructures	Theranostics with tracking; guided delivery concepts	Long-term retention and safety uncertainty	External fields or light; surface-chemistry dependent release	Surface chemistry and impurity control, clearance profiling, extended toxicology, justification of persistence risk	Imaging-enabled CNS delivery constructs; theranostic small molecules	[233,234,241]
Hybrid and biomimetic systems, corona-controlled designs	Multi-cargo and multi-function designs; layered targeting	CMC complexity and regulatory ambiguity	Layered diffusion and erosion plus pH or redox or enzyme triggers; optional external triggers	PAT monitoring, incoming material specs, control of surface functionalization and corona, batch fidelity	Angiopep-2 conjugated nanodrugs; dual-ligand lipid nanocarriers	[233,248,249]
Biogenic vesicles (exosomes, plant EVs, mimetics)	High biocompatibility; proteins and nucleic acids; abundant PDEVs	Batch variability, yield and purification, regulatory classification	Uptake and intracellular routing dependent release	Standardize source and isolation, potency assays, acceptable heterogeneity definition, scale-up and regulatory planning	Small molecules, proteins, nucleic acids; antioxidant and anti-inflammatory cargos	[234,240,250]

BBB, blood–brain barrier; CMC, chemistry, manufacturing, and controls; CNS, central nervous system; CPP, cell-penetrating peptide; EVs, extracellular vesicles; NLCs, nanostructured lipid carriers; NPs, nanoparticles; PAMAM, poly(amidoamine); PAT, process analytical technology; PDEVs, plant-derived extracellular vesicles; PEG, polyethylene glycol; PLGA, poly(lactic-co-glycolic acid); QbD, quality by design; siRNA, small interfering RNA; SLNs, solid lipid nanoparticles.

**Table 4:** RMT ligands for BBB delivery: binding/valency design levers, species caveats, and reported brain-exposure deltas. This table summarizes RMT ligand classes by target receptor, binding strength (K_d_), and valency/ligand density—key determinants of productive transcytosis versus endothelial sequestration and lysosomal routing. Species caveats flag translation pitfalls, including endogenous ligand competition (e.g., transferrin/insulin), regional and disease-dependent receptor expression, and limited rodent–human cross-reactivity. Δ brain exposure records fold-changes (AUC, K_p,brain_, or preferably K_p,uu_) versus matched non-targeted controls under comparable dosing and sampling windows. “NR” indicates values not specified in the current draft and to be completed during final reference curation.

Ligand/Target	K_d_ Range	Valency	Species Caveats	Δ Brain Exposure (Fold)	Notes	References
Transferrin (Tf) → TfR	NR in manuscript; productive “sweet spot” emphasized (avoid very high avidity)	Mono- to multivalent (ligand density-dependent)	Endogenous Tf competition; receptor expression varies by region/disease; rodent–human differences	NR; enter as fold vs. non-targeted control (specify metric: AUC, K_p,brain_, K_p,uu_)	Canonical BBB shuttle; format and density govern recycling vs. lysosomal routing; can be paired with parenchymal motifs	[291,292]
Anti-TfR antibodies/fragments/bispecific shuttles → TfR	NR in manuscript; affinity and epitope selection critical	Often monovalent/low-avidity formats preferred; bispecific designs common	Epitope-specific species cross-reactivity; high affinity can increase trapping; saturation effects	NR; report with dosing window and comparator	Design goal is efficient transcytosis with minimal TfR downregulation and reduced endothelial retention	[293,294,295]
Angiopep-2 → LRP1	NR in manuscript; ligand density and avidity tuning highlighted	Typically multivalent on nanocarriers; density optimized to avoid sequestration	LRP1 expression/context dependence; human relevance must be confirmed; tumor vs. healthy BBB differences	NR; report relative to non-targeted carrier	Widely used peptide shuttle for nanoparticles and conjugates; can support glioblastoma-directed constructs	[296,297,298]
ApoE-mimetic peptides → LDLR (±LRP1)	NR in manuscript; affinity window and release kinetics emphasized	Mono- or multivalent; avidity increases uptake but can increase trapping	Strong endogenous ApoE/LDL competition; lipid-state effects; species differences in lipoprotein biology	NR; specify endpoint (brain/plasma ratio, AUC)	Leverages lipoprotein trafficking; cleavable linkers and controlled valency can aid parenchymal release	[299,300]
Insulin/engineered IR ligands/anti-IR formats → IR	NR in manuscript; avoid receptor saturation	Low-avidity designs generally favored	Physiological ceiling and safety constraints (glucose homeostasis); high endogenous competition; species differences	NR; report alongside safety/tolerability	Attractive but constrained by homeostatic receptor function; format and dosing are decisive	[255]
Aptamers/alternative binders (e.g., TfR- or LRP1-binding) → RMT receptors	NR in manuscript; receptor-specific values to be inserted	Usually monovalent; multimerization possible	Cross-reactivity and epitope mapping required; stability in plasma and nuclease resistance differ by species	NR; populate with harmonized assay definitions	Modular alternatives to peptides/antibodies; can reduce immunogenicity but require robust CMC characterization	[291,301]

ApoE, apolipoprotein E; AUC, area under the curve; BBB, blood–brain barrier; CMC, chemistry, manufacturing, and controls; IR, insulin receptor; K_d_, dissociation constant; K_p,brain_, brain-to-plasma partition coefficient; K_p,uu_, unbound tissue-to-unbound plasma partition coefficient; LDL, low-density lipoprotein; LDLR, low-density lipoprotein receptor; LRP1, low-density lipoprotein receptor-related protein 1; NR, not reported; RMT, receptor-mediated transcytosis; Tf, transferrin; TfR, transferrin receptor.

**Table 5:** Alternative routes and device-enabled BBB modulation: evidence and risk–mitigation matrix for improving CNS exposure. This table summarizes alternative routes and device-enabled strategies to bypass or locally modulate the BBB when systemic nanocarriers or prodrugs are insufficient. Evidence level (preclinical to clinical use) is paired with key translational risks. Intranasal delivery enables olfactory/trigeminal access but is limited by variability and dose volume. FUS with microbubbles provides precise, reversible BBB opening requiring cavitation-aware control and imaging verification. Osmotic/chemical disruption increases permeability at the cost of safety and control. CED enables focal infusion but is invasive. External-field approaches remain exploratory. Where applicable, device-enabled BBB opening can be combined with systemically administered nanocarriers to create a pragmatic short-term combination paradigm.

Modality	Mechanism	Evidence Level	Clinical Status	Advantages	Risks/Mitigations	References
Intranasal nose-to-brain (sprays, gels, nanoemulsions)	Direct transport along olfactory and trigeminal pathways; reduced first-pass metabolism; mucoadhesion prolongs residence	Preclinical strong; early clinical emerging (context-dependent)	Used clinically for some CNS-active small molecules; delivery platforms under evaluation for neurodegeneration/oncology	Noninvasive; rapid onset potential; bypasses systemic dilution for suitable payloads; compatible with solubility-enabling formulations	High inter-individual variability (anatomy, mucociliary clearance); limited dose volume; nasal irritation—mitigate with device optimization, deposition mapping, mucoadhesive/in situ gels, and PK endpoints (AUC, K_p,uu_ when feasible)	[4,161,339]
Focused ultrasound (FUS) + microbubbles	Pulsed acoustic exposure drives stable cavitation-mediated mechanoporation and transient tight-junction/transport changes enabling local BBB opening	Robust preclinical; multiple early clinical studies	Clinical translation under way (MRI-guided protocols in neuro-oncology and neurodegeneration)	Spatially targeted, reversible opening; compatible with diverse payloads (small molecules, prodrugs, nanocarriers, biologics); enables region-specific dosing	Hemorrhage/edema risk with inertial cavitation; off-target opening—mitigate via cavitation monitoring (acoustic emissions/PCD), conservative parameter sets, MRI guidance, contrast-enhanced confirmation, and predefined abort thresholds	[154,340,341]
Osmotic BBB disruption (intra-arterial mannitol)	Hyperosmolar shrinkage of endothelial cells transiently widens tight junctions and increases permeability	Established concept; variable evidence by indication and protocol	Applied in select centers/indications; invasive and less commonly used than device-guided opening	Can increase delivery of otherwise excluded agents; compatible with intra-arterial co-administration	Poor spatial control; seizure/edema/hemorrhage risk; procedure-related risks—mitigate with stringent patient selection, hemodynamic monitoring, imaging surveillance, and avoidance of programs relying on nonspecific leak	[154,340,342]
Chemical permeability modulation (selected permeabilizers/co-solvents)	Transiently alters membrane integrity, tight-junction signaling, or transporter function to raise permeability	Limited to mixed; often preclinical or adjunctive	Not routine for broad CNS delivery; used cautiously as adjuncts in narrow settings	Potentially simple to implement; can be paired with systemic dosing when local devices are unavailable	Nonspecific barrier disruption and systemic toxicity; unpredictable PK and inflammation—mitigate with minimal-effective exposure, local delivery where possible, tight safety biomarkers, and preference for controllable modalities	[154,342,343]
Convection-enhanced delivery (CED)	Pressure-driven interstitial infusion via intracranial catheter achieves high local concentrations independent of BBB transport	Strong preclinical/clinical experience in focal indications	Clinical use and trials in neuro-oncology and focal CNS targets; procedure-dependent	High local dose; bypasses efflux and systemic barriers; controllable infusion profiles; suitable for macromolecules and particles	Invasive; catheter placement errors, reflux/backflow, heterogeneous distribution, infection—mitigate with image-guided planning, real-time distribution tracking, optimized cannula design, and sterility controls	[332,344,345]
External-field targeting/triggering (magnetic guidance; remote release)	Magnetic gradients concentrate magnetically responsive carriers; external fields can trigger release from stimuli-responsive constructs	Primarily preclinical; exploratory translation	Investigational; requires specialized hardware and long-term safety data	Adds spatiotemporal control without barrier-wide opening; can pair with imaging-enabled carriers for tracking	Uncertain long-term retention/clearance; heating and off-target accumulation; device standardization gaps—mitigate with biocompatible coatings, rigorous dosimetry, biodistribution/clearance studies, and conservative escalation	[4,154]

AUC, area under the curve; BBB, blood–brain barrier; CED, convection-enhanced delivery; CNS, central nervous system; FUS, focused ultrasound; MRI, magnetic resonance imaging; PCD, passive cavitation detection; PK, pharmacokinetics; K_p,uu_, unbound tissue-to-unbound plasma partition coefficient.

**Table 6:** Prodrug design playbook for BBB delivery: transporter-hijacking promoieties, brain-selective cleavage logic, and translation risks. This table condenses transporter-hijacking prodrug strategies into a practical design checklist for BBB delivery. It centers on key influx carriers (LAT1, GLUT1, MCT1), outlining common promoieties, linker chemistries, and brain-selective cleavage triggers that mitigate polarity, efflux, and instability while limiting premature systemic activation. Exposure gain is reported as fold-change versus parent or non-targeted controls using harmonized endpoints (brain AUC, K_p,brain_, preferably K_p,uu_), with “NR” for values not specified. Off-target risks include substrate competition, peripheral uptake, species differences, and unintended metabolite activity; notes emphasize K_m_/V_MAX_-aware design, linker stability, and assay/QC needs.

Transporter	Promoiety/Linker	Cleavage Trigger	Exposure Gain	Off-Target Risks	Notes	References
LAT1 (large neutral amino acid transporter)	L-amino acid promoieties (e.g., phenylalanine/leucine/tyrosine analogs); ester, amide, or carbamate linkers; optional self-immolative spacers	Brain-enriched esterases/peptidases; linker-enabled self-immolation after enzymatic trigger	NR (populate with fold-change in brain AUC, K_p,brain_ or K_p,uu_)	Competition with endogenous amino acids; saturation at high dose; peripheral uptake (gut, kidney); rodent–human affinity/epitope differences	Prefer moderate affinity to favor flux over trapping; design should be K_m_/V_MAX_-aware; verify brain-selective cleavage and low systemic conversion; include efflux liability screening for released parent	[401,402,403]
System L neutral amino acid transport (BBB uptake; LAT-family mediated)	Amino-acid precursor prodrug (4-chlorokynurenine; no external promoiety/linker)	Enzymatic bioconversion in CNS to 7-CKA via kynurenine-pathway transamination	Enhanced brain delivery vs. 7-CKA, enabling central glycine-site NMDA antagonism	Competition with dietary large neutral amino acids; peripheral metabolism yielding active/other metabolites; class-related CNS effects from glycine-site NMDA blockade	4-chlorokynurenine (AV-101) is a prodrug of 7-CKA; “facilitated brain uptake” demonstrated in perfusion studies	[404,405,406]
System L neutral amino acid transport (BBB uptake; LAT-family mediated)	Amino-acid precursor prodrug (4,6-dichlorokynurenine; no external promoiety/linker)	Enzymatic bioconversion in CNS to 5,7-DCKA via kynurenine-pathway transamination	Enhanced brain delivery vs. 5,7-DCKA, supporting higher-potency glycine-site NMDA antagonism	Similar System L competition liability; peripheral conversion; CNS tolerability risks typical of glycine-site NMDA antagonists	4,6-dichlorokynurenine is reported as a prodrug for 5,7-DCKA with facilitated brain uptake	[405,407,408]
Not transporter-targeted (SAR-driven KYNA analogs; prodrug-like exposure tuning)	KYNA scaffold with side-chain/ring substitutions (SZR series; e.g., methyl in SZR-72; C3 polar ring system in SZR-104)	None (active analogs; no enzymatic “unmasking”)	Improved BBB penetration and in vivo activity versus parent KYNA; SZR-72 neuroprotection and behavioral modulation; SZR-104 high BBB permeability with neuroprotection in sepsis; SZR-109 robust BBB entry with anti-inflammatory and anticonvulsant effects	Off-target kinase inhibition risk reported for SZR-105; broader off-target binding possible with structural diversification	Positions KYNA analogs as exposure-optimized leads rather than classic prodrugs; lead optimization may benefit from combination with IDO inhibitors to reshape KYN-pathway flux	[360,361,362]
GLUT1 (glucose transporter)	Glucose or glucosyl-like promoieties; O- or C-linked glycosides; carbonate/carbamate/ester linkers for release	Glycosidase-assisted unmasking (where applicable) and/or esterase-triggered cleavage of linkers; self-immolative release modules	NR (report with matched control and dosing window)	High peripheral distribution (erythrocytes/endothelium); competition with glucose; risk of rapid systemic cleavage; potential metabolic liabilities	Aim for productive transport without excessive binding; validate stability in plasma and nasal/intestinal matrices; monitor impact on glucose handling only where pharmacologically plausible	[401,409,410]
MCT1 (monocarboxylate transporter)	Monocarboxylate promoieties (e.g., lactate/pyruvate/acetate-like); ester linkers; soft-drug variants to tune logD	Carboxylesterase-mediated cleavage; pH/enzyme-sensitive linkers can bias release toward brain compartments	NR (capture as fold-change in brain exposure and unbound fraction when available)	Peripheral uptake (muscle, liver); competition with endogenous monocarboxylates; acidosis-related confounding in sensitive settings; species differences in transporter expression	Useful for polar acids/phenolics; quantify competition effects under physiological substrate levels; include brain-selective cleavage validation and metabolite profiling	[411,412,413]
Multiple SLCs (exploratory/case-by-case)	Nutrient-mimetic fragments matched to a selected transporter’s substrate space; modular linkers (esters/amides/carbamates)	Enzyme-labile trigger + self-immolative release (design-dependent)	NR (insert when transporter, affinity, and PK endpoints are specified)	Uncertain selectivity; off-target tissue uptake; unpredictable metabolism; model-to-human translation risk	Use only with strong transporter evidence (expression at BBB + uptake assays); pair with orthogonal confirmation (inhibitors/knockdown, saturability, competitive substrates)	[401,414,415]
Efflux-evading (non-transporter-hijacking) prodrugs	Mask H-bond donors/acceptors; increase logD modestly; promoieties that reduce recognition by P-gp/BCRP; soft-drug linkers	Systemic or brain esterases (must be tuned to avoid premature conversion)	NR (report brain exposure and safety vs. parent)	Premature systemic activation; altered distribution and toxicity; active metabolite formation; drug–drug interactions	Useful when influx carriers are not practical; requires early efflux screening (P-gp/BCRP) and rigorous metabolite ID; prioritize K_p,uu_ as decision endpoint	[415,416,417]

5,7-DCKA, 5,7-dichlorokynurenic acid; 7-CKA, 7-chlorokynurenic acid; AUC, area under the curve; BBB, blood–brain barrier; BCRP, breast cancer resistance protein; C3, carbon 3 position; CNS, central nervous system; GLUT1, glucose transporter 1; IDO, indoleamine 2,3-dioxygenase; K_m_, Michaelis constant; K_p,brain_, brain-to-plasma partition coefficient; K_p,uu_, unbound tissue-to-unbound plasma partition coefficient; KYN, kynurenine; KYNA, kynurenic acid; LAT1, large neutral amino acid transporter 1; logD, distribution coefficient; MCT1, monocarboxylate transporter 1; NMDA, N-methyl-D-aspartate; NR, not reported; P-gp, P-glycoprotein; PK, pharmacokinetics; QC; quality control; SAR, structure–activity relationship; SLCs, solute carrier transporters; System L, large neutral amino acid transport system (LAT-family mediated); SZR, SZR series KYNA analogue family; V_MAX_, maximum transport rate.

**Table 7:** Models-to-endpoints crosswalk for BBB delivery: throughput, predictive scope, artifacts, and best-fit development decisions. This crosswalk links commonly used BBB/CNS delivery models discussed in the manuscript to the endpoints they most reliably support. For each model, throughput is contrasted with predictive scope (barrier integrity, transporter effects, regional delivery, or human translation), and key artifacts that bias interpretation are flagged. The Best-fit decision column indicates where each approach is most informative in a development workflow—from early screening to go/no-go based on decision-grade brain exposure (e.g., K_p,uu_) and target engagement.

Model	Throughput	What It Predicts	Key Artifacts	Best-Fit Decision	References
Transwell BBB (endothelial mono-/co-culture; static TEER/permeability)	High	Relative permeability and gross barrier integrity; early ranking of formulations/prodrugs; qualitative efflux effects (context-dependent)	Static conditions; nonphysiologic shear; variable tight junction maturation; transporter expression drift; adsorption to plastics	Early screen and rank-order; eliminate non-starters before costly models	[499,500,501]
iPSC-derived BBB endothelium (Transwell)	Medium	More human-relevant tight junctions/transporters; better prediction of human-like permeability windows	Differentiation variability; batch effects; incomplete neurovascular unit (NVU) signaling unless co-cultured	Mid-stage confirmation of BBB-relevant transport and efflux liability	[502,503,504]
iPSC BBB organoids/spheroids (NVU-like)	Medium–low	3D cell–cell interactions, uptake and penetration trends; neuroinflammation-compatible testing	Size heterogeneity; diffusion limits; limited perfusion; measurement standardization gaps	Mechanism prioritization and safety/uptake profiling; compare targeting vs. non-targeting designs	[505,506,507]
Microfluidic BBB-on-chip (flow/shear; NVU co-culture)	Low–medium	Dynamic barrier responses under flow; transporter-mediated flux; inflammation-dependent permeability shifts	Device-to-device variability; bubble/absorption effects; complex operation; limited throughput	Late preclinical de-risking for mechanism and context dependence (inflammation, disease cues)	[508,509,510]
Rodent in vivo PK (brain + plasma; brain/plasma ratios)	Medium	System-level exposure, metabolism, distribution; initial signal of CNS delivery improvement	Species differences in BBB properties and transporters; confounding by vascular space and binding; anesthesia effects	Go/no-go based on integrated exposure; prioritize candidates for quantitative endpoints (K_p,uu_)	[499,503,511]
Rodent microdialysis (ISF sampling)	Low	Unbound interstitial exposure and time-course; closest preclinical readout to target-site pharmacology	Invasive; recovery calibration; regional restriction; limited to specialized setups	Decision-grade confirmation of CNS penetration (K_p,uu_-like inference) and PK/PD linkage	[499,503,511]
CSF sampling (preclinical/clinical)	Medium	Surrogate exposure trends when ISF is unavailable; supports translational sampling designs	CSF ≠ ISF; compartmental delays; protein binding differences; disease-state confounding	Clinical feasibility planning; supportive evidence alongside imaging or modeling	[499,503,511]
PET imaging (labeled payload or marker)	Low	Whole-brain/spatial distribution; target engagement surrogates; longitudinal kinetics in vivo	Radiolabel alters properties; metabolite signal; resolution limits; tracer-specific assumptions	Translation-facing biodistribution and engagement readouts; de-risk regional delivery claims	[504,512,513]
Non-human primate (NHP) studies	Very low	Closest approximation to human BBB transport and PK; de-risks scale and delivery paradigm	Cost/ethics; small n; limited disease modeling; procedural constraints	Preclinical-to-clinical bridge for top candidates and delivery devices/targeting ligands	[499,503,511]
Mechanistic PBPK/BBB models (incl. efflux and binding)	High (in silico)	Scenario testing; dose-to-exposure translation; integrates binding, efflux, and tissue partitioning	Parameter uncertainty; requires high-quality input data; model misspecification risk	Study design, endpoint selection, and translation planning; interpret CSF/ISF and imaging outputs	[499,503,511]

BBB, blood–brain barrier; CNS, central nervous system; CSF, cerebrospinal fluid; ISF, interstitial fluid; iPSC, induced pluripotent stem cell; K_p,uu_, unbound tissue-to-unbound plasma partition coefficient; NHP, non-human primate; NVU, neurovascular unit; PBPK, physiologically based pharmacokinetic; PD, pharmacodynamics; PET, positron emission tomography; PK, pharmacokinetics; TEER, transendothelial electrical resistance.

**Table 8:** Clinical snapshot of CNS delivery strategies in the manuscript: indication-by-modality overview, endpoints, and exposure evidence. This table provides a high-level clinical snapshot of the delivery modalities discussed in the manuscript, organized by representative CNS indications. For each entry, the development phase and primary clinical endpoint are summarized alongside the type of exposure evidence available (e.g., imaging-verified BBB opening, CSF/PK surrogates, or decision-grade brain exposure metrics when reported.

Indication	Modality	Phase	Primary Endpoint	Exposure Evidence	Status	References
Neurodegeneration (Alzheimer’s/Parkinson’s)	Focused ultrasound (FUS) + microbubbles	Phase I (as noted in manuscript)	Safety/tolerability; imaging-confirmed BBB opening	MRI guidance + contrast-enhanced confirmation; BBB resealing within hours (NR details)	Early clinical translation under way	[311,552,553]
Glioblastoma/focal CNS tumors	RMT-targeted nanocarriers (e.g., TfR/LRP1 ligands; Angiopep-2-type designs)	Preclinical → early clinical (NR)	Tumor response/progression metrics (NR)	Biodistribution/brain uptake signals; comparator vs. non-targeted carrier (NR)	Investigational; target/format-dependent	[230,554,555]
Glioblastoma/focal CNS tumors	Convection-enhanced delivery (CED)	Clinical use/trials (NR)	Local control and safety (procedure-specific)	High local concentration by direct interstitial infusion; distribution tracking (NR)	Procedure-dependent; used in specialized settings	[330,331,556]
Glioblastoma/focal CNS tumors	Liposomal curcumin (systemic lipid nanocarrier)	Phase Ib/IIa (early clinical)	Safety/tolerability; PK; exploratory imaging response	Systemic IV dosing with PK; imaging endpoints where available	Early clinical evaluation	[557,558,559]
Depression/neuropsychiatric disorders	Intranasal nose-to-brain formulations (sprays, gels, nanoemulsions)	Preclinical → early clinical signals (NR)	Symptom scales and tolerability (NR)	PK/PD signals; CSF or surrogate exposure where available (NR)	Emerging; high variability and formulation-sensitive	[339,560,561]
Broad CNS indications (adjunct permeability strategies)	Osmotic BBB disruption (intra-arterial mannitol)	Selective clinical application (NR)	Feasibility/safety; delivery enhancement (NR)	Increased permeability by protocol; exposure quantification variable (NR)	Invasive; limited use due to safety/control trade-offs	[327,562,563]
Exploratory/device-enabled targeting	External-field targeting/triggering (magnetic guidance; remote release)	Preclinical	Proof-of-concept delivery and safety	Tracking-enabled carriers; biodistribution and clearance studies (NR)	Exploratory; hardware and long-term safety gaps	[311,564,565]

BBB, blood–brain barrier; CED, convection-enhanced delivery; CNS, central nervous system; CSF, cerebrospinal fluid; FUS, focused ultrasound; LRP1, low-density lipoprotein receptor-related protein 1; MRI, magnetic resonance imaging; NR, not reported; PD, pharmacodynamics; PK, pharmacokinetics; RMT, receptor-mediated transcytosis; TfR, transferrin receptor.

## Data Availability

No new data were created or analyzed in this study.

## Abbreviations

The following abbreviations are used in this manuscript:

ABC	ATP-binding cassette
ApoE	apolipoprotein E
AUC	area under the curve
BBB	blood–brain barrier
BCRP	breast cancer resistance protein
CBD	cannabidiol
CED	convection-enhanced delivery
CMC	chemistry, manufacturing, and controls
CMT	carrier-mediated transport
CNS	central nervous system
CPP	cell-penetrating peptide
CQA	critical quality attribute
CSF	cerebrospinal fluid
CYP	cytochrome P450
EVS	extracellular vesicles
f_u,brain_	fraction of unbound drug in brain tissue
f_u,p_	unbound plasma fractions
FUS	focused ultrasound
GDNF	glial-derived neurotrophic factor
GLUT1	glucose transporter 1
GMP	good manufacturing practice
IPSC	induced pluripotent stem cell
IR	insulin receptor
ISF	interstitial fluid
K_m_	Michaelis constant
K_d_	dissociation constant
K_p,brain_	brain-to-plasma partition coefficient
K_p,uu_	unbound tissue-to-unbound plasma partition coefficient
K_p,uu,brain_	unbound brain-to-unbound plasma partition coefficient
KYNA	kynurenic acid
LAT1	large neutral amino acid transporter 1
LD	linear dichroism
LDL	low-density lipoprotein
LDLR	low-density lipoprotein receptor
LOGD	distribution coefficient
LRP1	low-density lipoprotein receptor-related protein 1
MCT1	monocarboxylate transporter 1
MCTS	monocarboxylate transporters
MRI	magnetic resonance imaging
MRPS	multidrug resistance-associated proteins
NHP	non-human primate
NLCS	nanostructured lipid carriers
NMDA	N-methyl-D-aspartate
NPS	nanoparticles
NR	not reported
NVU	neurovascular unit
P-gp	P-glycoprotein
PAMAM	poly(amidoamine)
PAT	process analytical technology
PBPK	physiologically based pharmacokinetic
PCD	passive cavitation detection
PD	pharmacodynamics
PDEVS	plant-derived extracellular vesicles
PDI	polydispersity index
PEG	polyethylene glycol
PET	positron emission tomography
PK	pharmacokinetics
pK_a_	acid dissociation constant
PKPD	pharmacokinetic–pharmacodynamic
PLGA	poly(lactic-co-glycolic acid)
QbD	quality by design
QTPP	quality target product profile
RMT	receptor-mediated transcytosis
RVG	rabies virus glycoprotein
SIRNA	small interfering RNA
SLCS	solute carrier transporters
SLNS	solid lipid nanoparticles
SULT	sulfotransferases
TAT	trans-activator of transcription
TEER	transendothelial electrical resistance
TF	transferrin
TFR	transferrin receptor
THC	Δ9-tetrahydrocannabinol
UGT	UDP-glucuronosyltransferases
VEGF	vascular endothelial growth factor
V_MAX_	maximum transport rate
